# Just Not That Into You: Experiences of Indifference Toward a Romantic Partner

**DOI:** 10.1177/01461672251410278

**Published:** 2026-01-08

**Authors:** Mirna Đurić, Francesca Righetti, Giulia Zoppolat, Iris K. Schneider

**Affiliations:** 1 Vrije Universiteit Amsterdam, Netherlands; 2 Amsterdam University Medical Center, Netherlands; 3 TUD Dresden University of Technology, Germany; 4 University of Cologne, Germany

**Keywords:** indifference, romantic relationships, well-being, interpersonal evaluations

## Abstract

Theoretical work has proposed that people can have four different patterns of interpersonal evaluations: mostly positive, mostly negative, ambivalent (both positive and negative), and indifferent (neither positive nor negative). Notably, indifference has been largely overlooked by empirical research, despite growing evidence that indifferent feelings can occur in romantic relationships. To address this gap, we examined the associations of feelings of indifference toward one’s romantic partner with relationship and personal well-being across four studies (*N* = 2,490), using cross-sectional and longitudinal data from couples and individuals in relationships. To do so, we developed the Subjective Interpersonal Indifference Scale and established it as a valid and reliable measure to assess this evaluation. Our findings showed that indifference toward a romantic partner is associated with lower relationship and personal well-being, both concurrently and longitudinally. Underlying these associations were higher feelings of boredom in the relationship, higher desire for attractive alternatives, and lower intimacy.

## Introduction

Among the countless stimuli that elicit emotions and evaluations in a person’s life, one’s romantic partner is commonly seen as a significant source of strong affective reactions (e.g., Clark & Finkel, 2005; Clark et al., 2001; Mikulincer & Shaver, 2005). Indeed, romantic partners are typically expected to elicit positive emotions (e.g., Baker et al., 2017; Boyes & Fletcher, 2007; Neff & Karney, 2003), although they are also notorious for provoking strong negative feelings, for example, when conflicts or transgressions occur (e.g., Finkel et al., 2002; Mikulincer & Shaver, 2005; Murray et al., 2003). However, experiencing an *absence* of emotions toward one’s romantic partner – in other words, indifference – may be perceived as especially non-normative and, thus, challenging to deal with. While previous studies have shown that a significant portion of individuals report feeling indifferent toward their romantic partner – ranging from 8–12% (Hsieh & Hawkley, 2018) to 11–18% (Windsor & Butterworth, 2010) and 15–22% (Fincham & Linfield, 1997) – research on this phenomenon remains notably scarce. How is experiencing indifference toward one’s romantic partner linked to individual and relationship outcomes?

Previous theoretical work and empirical research on constructs that are related to indifference suggest that a lack of emotions poses a serious threat to romantic relationships (Abbasi & Alghamdi, 2017), is very difficult to treat in couples therapy (Whisman et al., 1997), and, potentially, is a precursor of relationship dissolution (Righetti et al., 2026). Despite its potential significance, very few studies have empirically examined the distinct consequences of experiencing indifference toward a romantic partner. The limited research available has produced relatively inconclusive results and has not utilized a scale designed to measure this construct effectively. As a result, there is a lack of understanding of how and why this evaluative state might be influential for individuals and their relationships. Our work addresses this gap by making the following contributions: First, we provide a comprehensive investigation of the associations between experiencing indifference toward one’s romantic partner and relationship and personal well-being, utilizing both cross-sectional and longitudinal approaches. Second, we delve into the mechanisms that link indifference to well-being, providing critical insights into how indifference operates within romantic relationships and how it is associated with relationship dynamics. To do so, we introduce the Subjective Interpersonal Indifference Scale, a tool specifically designed to measure the awareness of feelings of indifference toward another person, in this case, a romantic partner, thereby enabling a more precise assessment of this important, yet underexplored, construct.

### Indifference in Romantic Relationships

According to the Evaluative Space Model (Cacioppo et al., 1997), people’s evaluations of attitude objects can be conceptualized along independent positive and negative dimensions (i.e., to what extent positive evaluations are present and to what extent negative evaluations are present). This also applies to evaluations of another person (Uchino et al., 2001). In this framework, the positive evaluative axis and the negative evaluative axis together create a bivariate space where people’s overall evaluative state toward another person will be determined by the simultaneous intensity of positive and negative evaluations. Specifically, one of four different evaluative states can arise, with varying intensity: People can regard another person mostly positively (i.e., holding high positive and low negative evaluations), mostly negatively (i.e., high negative and low positive evaluations), feel ambivalent toward them (i.e., high positive and high negative evaluations at the same time), or feel indifferent toward them (i.e., low positive and low negative evaluations at the same time). This framework has been increasingly applied in empirical studies of both close and non-close relationships (e.g., Bushman & Holt-Lunstad, 2009; Fingerman et al., 2004; Holt-Lunstad et al., 2007; Hsieh & Hawkley, 2018; Liu & Upenieks, 2021; Mattson et al., 2013; Methot et al., 2017; Reblin et al., 2010; Rogge et al., 2017; Uchino et al., 2013, 2014).

However, while ample research has investigated processes related to having mostly positive or mostly negative partner evaluations (for an overview, see Joel et al., 2020; Righetti et al., 2026; Ross et al., 2019), and a growing body of work has been studying ambivalence in romantic relationships (e.g., Birmingham et al., 2019; Righetti et al., 2020; Uchino et al., 2014; Zoppolat et al., 2023), limited research has specifically examined the fourth evaluative state: indifference. The few existing studies on this evaluative state have mainly investigated indifference toward a romantic partner using a categorical approach, in which people are either clustered to be in a relationship characterized by indifference versus in a relationship characterized by positivity, negativity, or ambivalence (e.g., Fincham & Linfield, 1997; Hsieh & Hawkley, 2018; Liu & Upenieks, 2021; Rogge et al., 2017; Windsor & Butterworth, 2010). This approach has yielded mixed results. For example, Hsieh and Hawkley (2018) report some evidence that wives (but not husbands) in indifferent marriages felt more lonely than those in mostly positive and ambivalent marriages. In contrast, Liu and Upenieks (2021) found that husbands (but not wives) reported lower happiness and health in indifferent than in mostly positive marriages. Furthermore, Fincham and Linfield (1997) found that wives’ reports of behaviors in indifferent marriages did not significantly differ from those in mostly positive marriages, while Rogge et al. (2017) found less positive interactions and no significant difference in negative interactions in indifferent relationships compared to mostly positive relationships. Consequently, it remains unclear how and why experiencing indifference might be linked to people’s well-being in romantic relationships. Furthermore, the subjective experience of indifference remains unexplored, due to the absence of measures that capture the *awareness* and *degree* of people’s indifferent feelings, rather than simply categorizing them as being in an indifferent relationship vs. not.

Previous research has also explored constructs that are closely related to indifference from a theoretical perspective. For example, Barry et al. (2008) have examined *romantic disengagement*, defined as a lack of emotions toward one’s partner, accompanied by increasing cognitive and behavioral distancing from them. Romantic disengagement has been found to be negatively associated with relationship satisfaction, commitment, intimacy, and passion (Barry et al., 2008). Another example is *disaffection*, defined as an increasing lack of emotions, attachment, and caring in the relationship (Kersten, 1990), which was also found to be linked to lower marital satisfaction and closeness (Kayser, 1996). Although these lines of research have produced important insights into processes in relationships that are, to a certain extent, related to indifference, it remains unclear how constructs like disengagement or disaffection fit within the four patterns of partner evaluations. In fact, these constructs, and their corresponding measurement tools, capture elements of indifference, but they also include negative emotions toward the partner, as well as negative motivational and behavioral outcomes – such as withdrawal, destructive behaviors, and a general lack of care for one’s partner – which may be, but are not necessarily, consequences of indifference (Righetti et al., 2026). In contrast, studies seeking to investigate indifference as conceptualized by the Evaluative Space Model (Cacioppo et al., 1997) and theoretical frameworks of interpersonal evaluations (e.g., Righetti et al., 2026; Uchino et al., 2001) should aim to capture indifference directly – as having neither strong positive nor strong negative feelings toward a partner – without confounding it with negative affect, negative behaviors, general disengagement, or its potential consequences (Righetti et al., 2026). To fill this gap, the present work develops a scale that offers a comprehensive and specific assessment of interpersonal indifference without containing items that measure negative evaluations, motivations, or behaviors. Importantly, we examine *subjective* experiences of indifference – that is, people’s awareness and reported degree to which they experience indifferent feelings toward their partner (see also Wilson, 2022), which is likely to be especially consequential for their well-being, as indicated by research on subjective experiences of other complex evaluative states such as ambivalence (e.g., van Harreveld et al., 2015; Zoppolat et al., 2023).^1^ Using this measure, we conduct a comprehensive investigation of the unique consequences of feeling indifferent toward one’s romantic partner.

### Associations With Well-Being and Underlying Mechanisms

We set out to investigate the associations of feeling indifferent toward one’s partner with key indicators of relationship and personal well-being, and the mechanisms that might explain these associations. Theoretical work has argued that people feel indifferent toward others when they neither provide meaningful rewards, which typically evoke positive evaluations, nor impose significant costs, which are likely to elicit negative evaluations (Gasper et al., 2019; Kelley & Thibaut, 1978; Righetti et al., 2026). Importantly, such a dynamic is likely to be experienced as non-normative in the context of a romantic relationship and interpreted as a sign that the relationship is unfulfilling. This is significant as romantic relationships are expected to be among the primary sources of people’s need fulfillment, fulfilling needs such as those for security, belonging, esteem, and even self-growth and self-actualization (e.g., Baumeister & Leary, 1995; Finkel et al., 2014; Murray et al., 2006). When romantic partners cannot fulfill these needs, even if there are no actual conflicts or overt negativity in the relationship, people are likely to find this unsettling, as they expect more gratification from a romantic relationship (e.g., Finkel et al., 2014; Le & Agnew, 2001). Furthermore, building on appraisal theories of emotion (for a review, see Moors et al., 2013), which posit that emotions arise in response to stimuli that are seen as personally relevant, indifference (as a lack of emotion) would imply that the partner has been appraised as unimportant (Cohen-Chen et al., 2022; van Kleef, 2016). Such an appraisal and resulting experience is likely to be regarded as non-normative in a romantic relationship, since a romantic partner is typically expected to be a highly significant figure in a person’s life who elicits strong emotions (e.g., Clark et al., 2001; Mikulincer & Shaver, 2005), both positive (e.g., through support, sexual fulfillment) and occasional negative (e.g., through conflict, misunderstanding), making the absence of any strong affect toward them feel abnormal and substantially undesirable. Thus, we hypothesize that experiencing feelings of indifference toward one’s own romantic partner will negatively contribute to relationship and personal well-being. In support of this idea, previous research has indeed provided some, although not entirely consistent, indications that indifference might be associated with higher loneliness (Hsieh & Hawkley, 2018) and lower happiness and health (Liu & Upenieks, 2021). Furthermore, studies on related constructs, such as romantic disengagement and disaffection, have also shown negative correlations with relationship quality (e.g., Barry et al., 2008; Kayser, 1996), although, as mentioned before, measures of these constructs often also involve some explicit negativity toward the partner.

To further understand why there should be a negative association between feelings of indifference toward one’s partner and well-being in the context of a romantic relationship, we investigated three key potential underlying mechanisms. First, we anticipated that indifference toward a romantic partner would be associated with higher feelings of *boredom* in the relationship. Namely, feelings of indifference may indicate that the partner is not a source of interest (Gasper et al., 2019), does not provide particular rewards or costs (Righetti et al., 2026), and is therefore unstimulating, contributing to the experience of boredom in the relationship (Harasymchuk & Fehr, 2010, 2012, 2013). In turn, we anticipated relational boredom to be associated with lower relationship and personal well-being, as previous studies have shown its links to lower relationship quality both cross-sectionally (Aron et al., 2000; Harasymchuk & Fehr, 2012) and longitudinally (Tsapelas et al., 2009), and boredom, more broadly, has been linked to lower personal well-being (e.g., Goldberg et al., 2011).

Along similar lines, we expected that people who feel indifferent toward their romantic partner would increasingly devote their *attention to other domains* of their life, outside of the current relationship. Indeed, since feelings of indifference signal that the current partner is not particularly worthy of attention (Gasper et al., 2019) or able to fulfill needs, people may redirect their motivational resources toward other, potentially more fruitful, pursuits (Righetti et al., 2026). Specifically, when experiencing indifference toward their current partner, people might experience higher desire for attractive alternatives (i.e., other possible romantic partners) and/or invest more energy into their work, hobbies, or other people in a non-romantic context (e.g., friends, family) that may be better able to fulfill their personal needs. Ultimately, devoting closer attention to other domains is expected to be linked to lower relationship well-being in one’s current romantic relationship, as desiring attractive alternatives (e.g., Zoppolat et al., 2022) and investing in other domains at the expense of the relationship (Kumashiro et al., 2008) can be detrimental for relationship quality.

Finally, we expected that indifferent individuals would experience a lower sense of *intimacy* in their relationship. As the partner does not provide particular rewards or costs, people should experience a decreased desire to engage with them and to be close to them, reducing feelings of intimacy (e.g., Laurenceau et al., 2005; Lippert & Prager, 2001; van den Broucke et al., 1995). We expected that this lack of intimacy would, in turn, lead to lower relationship and personal well-being, as intimacy is one of the key aspects of relationship quality (Fletcher et al., 2000; Hassebrauck & Fehr, 2002; Joel et al., 2020) and has also been shown to contribute to people’s personal well-being (e.g., Poucher et al., 2022; Prager & Buhrmester, 1998).

### The Present Research

Although it has been theoretically recognized that people can have indifferent evaluations toward their romantic partner (e.g., Righetti et al., 2026; Uchino et al., 2001), and studies have indicated that indifference is indeed experienced in romantic relationships (e.g., Fincham & Linfield, 1997; Hsieh & Hawkley, 2018), empirical research has largely neglected this evaluative state. In this work, we seek to expand the limited and mixed previous findings on the experience of indifference toward a romantic partner by providing a comprehensive investigation into the associations between feelings of indifference toward one’s romantic partner and key psychological outcomes and by introducing a novel instrument designed to measure such indifferent feelings specifically.

We hypothesized that feelings of indifference toward a romantic partner would be associated with lower relationship well-being (i.e., lower relationship satisfaction, commitment, trust, and higher thoughts of breaking up^2^) and personal well-being (i.e., lower life satisfaction and higher depression). In addition to these outcomes for which we had a priori predictions, we examined the associations between indifference and stress in an exploratory manner, since indifference toward a romantic partner could either be linked to higher stress (because a lack of emotion is a non-normative experience in romantic relationships and people may feel pressure to feel differently; e.g., Clark et al., 2001; Mikulincer & Shaver, 2005) or have no link to stress (because indifference, as opposed to positivity, negativity, and ambivalence, is a relatively low-arousal and de-activating evaluative state; Righetti et al., 2026; Schneider et al., 2016). We further examined several mechanisms responsible for the associations between indifference and well-being. Specifically, the associations between indifference and relationship well-being outcomes might arise because of higher relational boredom and attention to other domains (work, hobbies, other people such as friends and family, and attractive alternatives), and because of lower intimacy. Similarly, the associations between indifference and personal well-being outcomes might arise because of higher relational boredom and lower intimacy (Figure 1 shows the proposed conceptual models). Each of the hypothesized mechanisms and outcomes was tested in a separate model.^3^

**Figure 1.**
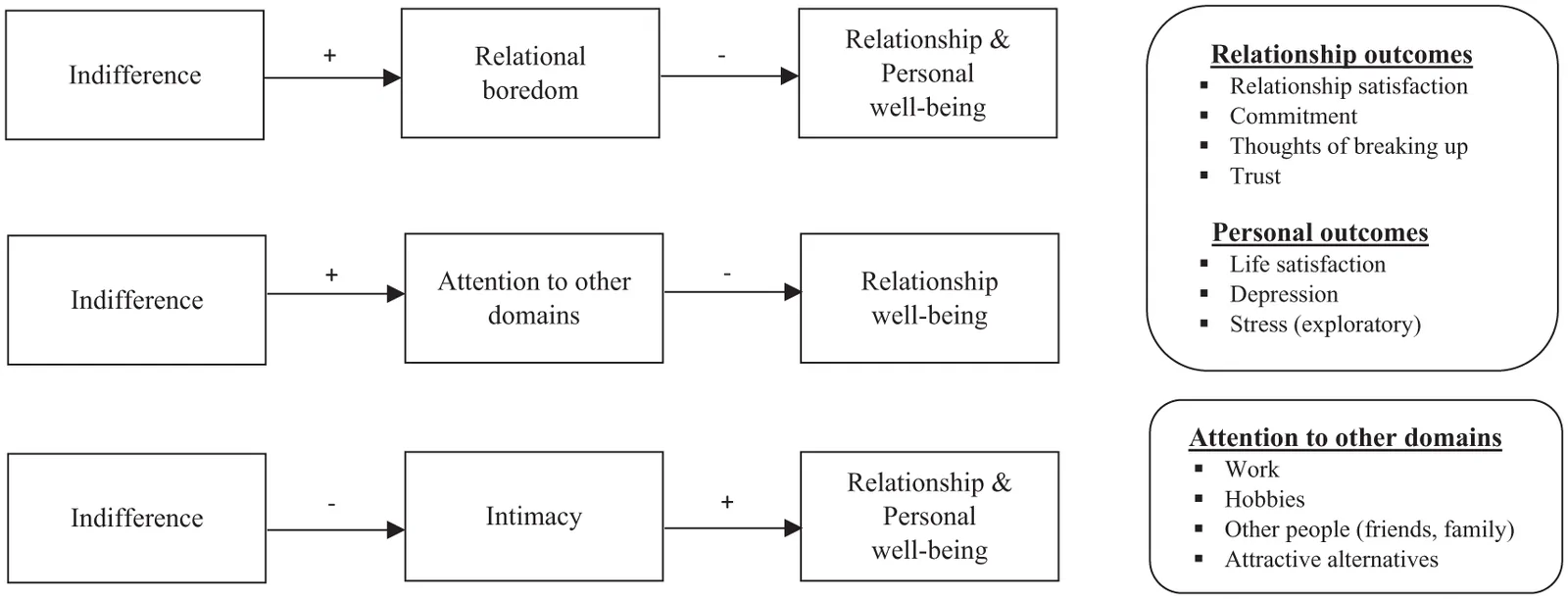
Conceptual models for the associations of indifference with relationship and personal well-being through relational boredom, intimacy, and attention to other domains. *Note.* Each of the relationship and personal well-being indices was tested as an outcome in a separate model.

We tested our hypotheses across four studies. The Pilot Study (reported in Supplemental Material^4^) provided an initial test of the hypotheses in a sample of individuals involved in a romantic relationship (*N* = 559) recruited on Prolific. In two pre-registered studies, Study 1 (*N* = 591) and Study 2 (*N* = 980), we developed the Subjective Interpersonal Indifference Scale and validated it in a sample of individuals involved in a romantic relationship. In those studies, we also used the scale to test our full set of hypotheses regarding the associations of indifference with relationship and personal well-being, and the underlying mechanisms. Finally, in Study 3, we replicated our main findings in a sample of romantic couples (*N* = 360 individuals involved in a romantic relationship) cross-sectionally and longitudinally over the span of 3 years.^5^ Across these four studies, we aimed to ensure that the examined associations were specific to indifference and not caused by any presence of negative partner evaluation. Namely, since devaluing one’s partner is known to have negative links with well-being (see e.g., Joel et al., 2020; Righetti et al., 2026), we employed a stringent statistical approach to account for the influence that negative feelings toward one’s partner might have on the examined associations. Specifically, in all analyses, we controlled for one’s general (d)evaluation of their partner, following the approach commonly used in studies on ambivalent partner evaluations, where it is also important to separate the effects of interest from those of negative partner evaluations (e.g., Surjadi et al., 2023; Uchino et al., 2014; Zoppolat et al., 2022, 2023). Thus, we report the *unique* associations of indifference with the examined outcomes and underlying mechanisms. Table 1 shows an overview of variables included in Studies 1 to 3, and Table 2 shows information about the samples, with details about sensitivity analyses reported in the Supplemental Material.

**Table 1.** Overview of Variables Across Studies 1–3.

Study	Indifference measure	Relationship outcomes	Personal outcomes	Mechanisms	Control variable
Study 1	Subjective Interpersonal Indifference Scale (8 items)	Relationship satisfaction Commitment Thoughts of breaking up Trust	Life satisfaction Stress Depression	Relational boredom	General partner (d)evaluation
Study 2	Subjective Interpersonal Indifference Scale (8 items)	Relationship satisfaction Commitment Thoughts of breaking up Trust	Life satisfaction Stress Depression	Relational boredom Intimacy Attention to other domains	General partner (d)evaluation
Study 3	Subjective Interpersonal Indifference Scale (short 3-item version)	Relationship satisfaction Commitment Thoughts of breaking up Trust	Life satisfaction Stress Depression	Relational boredom Intimacy Attention to other domains	General partner (d)evaluation

*Note.* Details about the Pilot Study are reported in the Supplemental Material.

**Table 2.** Overview of Sample Characteristics and Exclusions Across Studies 1–3.

Study/sample characteristic	Study 1	Study 2	Study 3
Final *N*	591 individuals	980 individuals	Baseline: 360 individuals Follow-up 1: 292 individuals Follow-up 2: 249 individuals Follow-up 3: 254 individuals Follow-up 4: 247 individuals Follow-up 5: 229 individuals Follow-up 6: 222 individuals
Original *N* and participation criteria	600 participants in relationships for at least 4 months, reside in the United States or the United Kingdom, passed at least two out of three attention checks	1,000 participants in relationships for at least 4 months, reside in the United States or the United Kingdom, passed at least two out of three attention checks	190 couples (i.e., 380 individuals) in relationships for at least 4 months, reside in the Netherlands
Exclusion criteria	9 failed honesty check	16 failed honesty check 3 due to relationship duration 1 due to not following instructions	*At baseline*: 9 failed at least two out of three attention checks, 5 withdrew from the study, 6 had technical difficulties *Across follow-up sessions*: Responses excluded if failed at least two attention checks, displayed low effort in survey, completed survey too late, had technical difficulties, or ended the relationship
Compensation	1.50 GBP (in accordance with Prolific payment regulations)	1.20 GBP (in accordance with Prolific payment regulations)	Up to 110 EUR in total
Gender	57.4% women, 42.3% men, 0.2% selected option “other” for gender, and 0.2% preferred not to answer	60.0% women, 39.2% men, 0.5% selected option “other” for gender, and 0.3% preferred not to answer	51.7% women, 48.3% men
Age	*M* = 43.9, *SD* = 13.5, range 18–78	*M* = 42.4, *SD* = 13.1, range 19–78	*M* *=* 38.7, *SD* = 14.6, range 18–75
Relationship duration	*M* = 186.6 months/15.6 years, *SD* = 156.6 months/13.1 years, range 4–660 months/55.0 years	*M* = 179.6 months/15.0 years, *SD* = 144.1 months/12.0 years, range 4–690 months/57.5 years	*M* = 149.4 months/12.5 years, *SD* = 147.9 months/12.3 years, range 4.5–651 months/54.3 years
Other demographic information (if available)	98.8% residing in the United Kingdom, 0.8% residing in the United States, 0.2% selected option “other” for country of residence, 0.2% preferred not to answer 88.0% living together with partner 57.2% married	95.5% residing in the United Kingdom, 4.2% residing in the United States, 0.3% preferred not to answer 88.0% living together with partner 59.6% married	82.8% living together with partner 42.5% married
Smallest detectable effect size at 0.80 power (sensitivity)	*f*^2^ = 0.013	*f*^2^ = 0.008	0.006 < *f*^2^ < 0.019

*Note.* The participants additionally excluded because of relationship duration in Study 2 were originally included into the samples because they passed the relationship duration filter on Prolific, but later in the survey self-reported a relationship duration below 4 months (see also pre-registration). Furthermore, we note that we did not pre-register excluding participants based on not following instructions in Study 2 as we did not anticipate this issue; however, we deemed it necessary to exclude such participants for the integrity of the data. Characteristics of the Pilot Study sample are reported in the Supplemental Material.

### Transparency and Openness

We report all relevant data exclusions and manipulations. The measures, analysis syntax/code, codebook, and pre-registrations corresponding to the individual studies are available on the project’s OSF page: https://osf.io/mfuz2/overview?view_only=f2aa6fef9bf344db803932e6ab33eba7. Data for the Pilot Study, Study 1, and Study 2 are also available on the OSF page, while data for Study 3 are not publicly available due to ethical regulations determined by the host institution (phrasing of the informed consent form and the sensitivity of dyadic data). Data collection for the Pilot Study, Study 2, and Study 3 was part of larger studies about romantic relationships. A list of measures included in each of the studies but not used for the present analyses is available in the Supplemental Material. We received approval from the Ethical Review Board at the host institution for all studies, and all participants provided informed consent prior to starting the study. We report all pre-registered analyses in the manuscript or Supplemental Material, and clearly mark deviations from the pre-registered analysis plans.

## Study 1

### Method

#### Participants and Procedure

We recruited participants on Prolific (see Table 2 for sample details). They filled out the online questionnaire containing the key measures described below, as well as demographic questions. They were then debriefed and paid for their participation. The study design, planned sample size, exclusion criteria, and analysis plan were pre-registered at https://osf.io/pcexk/overview?view_only=3ef7a6032d614d3393ce52cfb51f3a6f.

#### Measures

All measures were rated on a 7-point scale (1 = *Not at all/Do not agree at all*; 7 = *Completely/Completely agree*), unless noted otherwise.

##### Indifference

We initially generated 14 items that were aligned with the construct to measure *indifference* toward a romantic partner. The full set of items can be seen in Table 3. They were presented to participants in randomized order, with an introductory text: “To what extent do the following statements apply to you? Please respond as honestly as possible, there are no right or wrong answers. When giving your answers, please respond to the entire statement, not just parts of it. If you agree with only one half of the statement but not the other half, then you should indicate that the statement does not apply to you. For example, in the statement ‘*I do not feel much disappointment nor much excitement toward my partner*’, if you do not feel disappointment but you do feel excitement toward your partner, then you should reply that this statement does not apply to you.” Participants rated the items on a 7-point scale (1 = *Does not apply to me at all*, 7 = *Completely applies to me*). The factor loadings, means and standard deviations, and corrected item-total correlations for each of the items can be seen in Table 3, with the items retained in the final version of the Subjective Interpersonal Indifference Scale indicated in bold. The details of scale development and testing of its psychometric properties can be found in the Supplemental Material.

**Table 3.** Initial Pool of Items for the Subjective Interpersonal Indifference Scale With the Corresponding Factor Loadings for the One-Factor Structure, Means, Standard Deviations, and Corrected Item-Total Correlations.

Item	Factor loading	*M (SD)*	Corrected item-total correlation
**1. I do not feel strong emotions toward my partner at all (neither positive nor negative).**	.792	1.81 (1.33)	.76
**2. I feel a bit numb toward my partner.**	.767	1.74 (1.27)	.73
**3. I feel a bit indifferent toward my partner.**	.816	1.83 (1.39)	.78
4. When I think about my partner, my thoughts are neither very negative nor very positive.	.711	2.17 (1.60)	.69
5. I do not feel much disappointment nor much excitement toward my partner.	.669	2.08 (1.54)	.65
6. My partner is not a source of joy for me but they are also not a frustration.	.630	1.98 (1.48)	.61
**7. I find it difficult to feel strong emotions toward my partner.**	.788	1.93 (1.44)	.76
8. I feel intense emotions toward my partner.*	.712	2.54 (1.57)	.68
9. When I think about my partner, I feel powerful emotions.*	.738	2.51 (1.52)	.71
10. I have strong feelings toward my partner.*	.769	2.02 (1.38)	.74
**11. Both the negative and positive feelings that I have toward my partner are rather weak.**	.713	1.97 (1.40)	.69
12. I do not particularly highly value my partner but I also do not look down on them.	.668	1.76 (1.32)	.65
13. I do not have an extremely positive or an extremely negative opinion of my partner.	.652	2.27 (1.76)	.64
**14. I feel neutral toward my partner.**	.715	1.85 (1.38)	.69

*Note.* Items with an asterisk (*) are reverse-scored. Items in bold were retained in the final version of the scale.

##### Relationship Well-Being

To investigate criterion validity for the new indifference scale, we measured the key relationship and personal well-being outcomes that we hypothesized about. Namely, as indexes of relationship well-being, we measured *relationship satisfaction* using four items (Rusbult et al., 1998; e.g., “I feel satisfied with our relationship”), *commitment* using a 7-item scale (Rusbult et al., 1998; e.g., “I am committed to maintaining my relationship with my partner”), *thoughts of breaking up* using four items adapted from Booth et al. (1983) and Impett et al. (2010; e.g., “Have you ever seriously suggested the idea of breaking up?”; 1 = *Never*, 7 = *Every day*), and *trust* using a 12-item version (see Finkenauer et al., 2009; Schneider et al., 2011) of the trust scale developed by Rempel et al. (1985; e.g., “I have found that my partner is very dependable, especially when it comes to things which are important to me”).

##### Personal Well-Being

As indexes of personal well-being we measured *life satisfaction* using three items from the Satisfaction With Life Scale (Diener et al., 1985; e.g., “I am satisfied with my life”) and *stress* using the corresponding subscale of the Depression, Anxiety, and Stress Scale (DASS-21; Lovibond & Lovibond, 1995; e.g., “I found it difficult to relax”) rated on a 4-point scale (0 = *Did not apply to me at all*, 3 = *Applied to me very much or most of the time*).

##### Mechanism and Control Variable

As a mechanism, we measured *relational boredom* using the 15-item Relational Boredom Scale (Harasymchuk & Fehr, 2012), which asked participants how well several attributes described their relationship (e.g., “boring”). Finally, we measured participants’ *general partner (d)evaluation* using a 5-item scale (see e.g., Larson et al., 2022; Zoppolat et al., 2022), with an example item: “I like my partner very much.”

#### Analytic Strategy

To investigate the criterion validity of the new indifference scale and test our main hypotheses, we tested a separate linear regression model for each of the outcome variables (relationship outcomes: relationship satisfaction, commitment, thoughts of breaking up, trust; personal outcomes: life satisfaction; stress was examined exploratorily). In these models, next to indifference as the main predictor of interest, we included general partner (d)evaluation as a control variable. In line with our other studies, we also examined relational boredom as a mediator in the associations between indifference and relationship and personal well-being.^6^ To test the mediation hypotheses, we applied a combination of the joint-significance approach and indirect effect testing (Yzerbyt et al., 2018), where a hypothesis was considered supported if both the *a* and *b* paths in the mediation model were statistically significant (MacKinnon et al., 2002) and if the 95% confidence interval for the indirect effect estimate did not contain 0. We constructed this confidence interval with 5,000 bootstrap samples, using the PROCESS macro v. 4.2 for SPSS (Model 4; Hayes, 2022).

### Results

Table 4 shows the descriptive statistics, bivariate correlations, and reliability indices for key variables in Study 1.

**Table 4.** Means, Standard Deviations, Bivariate Correlations, and Cronbach’s Alpha for Key Variables in Study 1.

Variable	*M* (*SD*)	1.	2.	3.	4.	5.	6.	7.	8.	9.
1. Indifference	1.96 (1.11)	.92								
2. General partner (d)evaluation	6.20 (1.09)	−.78***	.96							
3. Relational boredom	2.72 (1.24)	.78***	−.84***	.95						
4. Relationship satisfaction	5.56 (1.37)	−.75***	.83***	−.88***	.95					
5. Commitment	6.34 (1.07)	−.69***	.82***	−.74***	.74***	.91				
6. Thoughts of breaking up	2.15 (1.13)	.55***	−.61***	.70***	−.69***	−.62***	.89			
7. Trust	5.72 (1.20)	−.66***	.76***	−.77***	.76***	.69***	−.71***	.93		
8. Life satisfaction	4.90 (1.53)	−.44***	.51***	−.58***	.64***	.47***	−.49***	.52***	.95	
9. Stress	0.92 (0.77)	.22***	−.30***	.38***	−.35***	−.26***	.36***	−.38***	−.48***	.93

*Note.* The Cronbach’s alpha for each scale is displayed on the diagonal.

*** p < .001.

#### Associations of Indifference With Relationship and Personal Well-Being

To examine criterion validity of the new scale, as well as test our main hypotheses, we tested associations of indifference as measured by the Subjective Interpersonal Indifference Scale with key relationship and personal well-being outcomes (i.e., total effects), above and beyond general partner (d)evaluation. The results are shown in Table 5. When it comes to relationship well-being, the results showed that indifference, as measured by the Subjective Interpersonal Indifference Scale, was negatively associated with relationship satisfaction, commitment, and trust, and positively associated with thoughts of breaking up. In terms of personal well-being, indifference, as measured by the Subjective Interpersonal Indifference Scale, was negatively associated with life satisfaction. We did not find a significant association between indifference and stress.

**Table 5.** Results of Testing the Total Effects of Indifference on Relationship and Personal Outcomes, Controlling for General Partner (D)Evaluation Across Studies.

Outcome	Predictor	Study 1				Study 2				Study 3 (cross-sectional)				Study 3 (longitudinal)			
		*b*	*SE*	*t*	*p*	*b*	*SE*	*t*	*p*	*b*	*SE*	*t*	*p*	*b*	*SE*	*t*	*p*
Relationship satisfaction	Indifference	**−0.31**	**0.04**	**−7.05**	**<.001**	**−0.20**	**0.03**	**−7.09**	**<.001**	**−0.15**	**0.01**	**−11.37**	**<.001**	**−0.05**	**0.02**	**−2.31**	**.021**
	General partner (d)evaluation	0.80	0.05	17.95	<.001	0.91	0.03	32.75	<.001	0.92	0.03	35.90	<.001	0.17	0.05	3.20	.001
Commitment	Indifference	**−0.14**	**0.04**	**−3.72**	**<.001**	**−0.14**	**0.03**	**−5.49**	**<.001**	**−0.08**	**0.01**	**−6.26**	**<.001**	**−0.06**	**0.02**	**−3.42**	**<.001**
	General partner (d)evaluation	0.70	0.04	18.72	<.001	0.61	0.03	23.53	<.001	0.57	0.03	22.98	<.001	0.13	0.04	3.37	<.001
Thoughts of breaking up	Indifference	**0.18**	**0.05**	**3.44**	**<.001**	**0.15**	**0.04**	**4.38**	**<.001**	**0.12**	**0.02**	**7.68**	**<.001**	**0.04**	**0.02**	**2.29**	**.022**
	General partner (d)evaluation	−0.49	0.05	−9.13	<.001	−0.62	0.04	−17.68	<.001	−0.36	0.03	−11.19	<.001	−0.12	0.04	−3.18	.002
Trust	Indifference	**−0.20**	**0.05**	**−4.23**	**<.001**	**−0.08**	**0.03**	**−2.70**	**.007**	**−0.09**	**0.01**	**−7.76**	**<.001**	**−0.03**	**0.01**	**−2.33**	**.020**
	General partner (d)evaluation	0.68	0.05	14.38	<.001	0.68	0.03	22.60	<.001	0.49	0.02	21.63	<.001	0.07	0.03	2.38	.018
Life satisfaction	Indifference	**−0.16**	**0.08**	**−2.06**	**.039**	**−0.15**	**0.05**	**−3.23**	**.001**	**−0.10**	**0.02**	**−5.65**	**<.001**	−0.03	0.02	−1.39	.166
	General partner (d)evaluation	0.59	0.08	7.31	<.001	0.62	0.05	13.03	<.001	0.34	0.04	9.63	<.001	0.04	0.05	0.99	.323
Depression	Indifference	–	–	–	–	**0.12**	**0.03**	**4.81**	**<.001**	**0.09**	**0.01**	**8.26**	**<.001**	**0.04**	**0.01**	**2.75**	**.006**
	General partner (d)evaluation	–	–	–	–	−0.16	0.03	−6.10	<.001	−0.05	0.02	−2.09	.037	−0.01	0.03	−0.35	.729
Stress	Indifference	−0.03	0.04	−0.60	.546	**0.08**	**0.03**	**2.52**	**.012**	**0.07**	**0.01**	**5.56**	**<.001**	0.03	0.02	1.71	.088
	General partner (d)evaluation	−0.23	0.05	−5.13	<.001	−0.13	0.03	−4.39	<.001	−0.10	0.03	−3.75	<.001	−0.04	0.03	−1.17	.241

*Note.* The significant total effects of indifference are indicated in bold. Dashes indicate that the respective effect was not tested in a given study.

#### Relational Boredom as a Mechanism

Furthermore, in line with our remaining studies, we tested whether relational boredom mediates the associations between indifference and relationship and personal well-being (i.e., indirect effects), above and beyond general partner (d)evaluation. The results showed that feelings of indifference toward one’s partner were associated with relationship well-being indices via higher relational boredom – specifically, with lower relationship satisfaction (indirect effect *ab* = −0.242; *SE* = 0.034; 95% CI [−0.312, -0.177]), commitment (*ab* = −0.050; *SE* = 0.018; [−0.087, −0.017]), and trust (*ab* = −0.147; *SE* = 0.026; [−0.201, −0.100]), as well as with higher thoughts of breaking up (*ab* = 0.203; *SE* = 0.032; [0.143, 0.270]). Indifference was also associated with personal well-being indices via higher relational boredom – specifically, with lower life satisfaction (*ab* = −0.239; *SE* = 0.041; [−0.324, −0.163]) and higher stress (*ab* = 0.121; *SE* = 0.023; [0.080, 0.169]).

### Discussion

In Study 1, we developed a new scale to measure interpersonal indifference, specifically applied to the experience of indifference toward a romantic partner, and sought to test our hypotheses regarding the associations between indifference and relationship and personal well-being. The scale showed satisfactory reliability, as indicated by a Cronbach’s alpha of .92, and satisfactory validity, as evidenced by a one-factor structure and indicators of convergent and discriminant (see Supplemental Material), as well as criterion validity. Indeed, indifference toward a romantic partner was associated as hypothesized with indices of relationship well-being (lower relationship satisfaction, commitment, and trust, as well as higher thoughts of breaking up) and with indices of personal well-being (lower life satisfaction). Higher relational boredom was found to be underlying these associations. Importantly, these findings held when controlling for general partner (d)evaluation, showing that they were unique to indifference rather than being driven by simply devaluing one’s partner. Subsequently, in Study 2, we used this scale to replicate the main associations between indifference and well-being indexes and test two additional mechanisms: more attention to domains outside of the relationship and lower intimacy.

## Study 2

### Method

#### Participants, Procedure, and Analytic Strategy

We recruited participants on Prolific (see Table 2 for sample details). Participants filled out the online questionnaire that included the described measures as well as demographic questions. Upon completion, they were debriefed and paid for their participation. The study design, planned sample size, exclusion criteria, and analysis plan were pre-registered at https://osf.io/j58yx/overview?view_only=8bff1b1b30fd4f7c9fea87424dc29174. We tested the hypotheses using the same analytic approach as described for testing criterion validity in Study 1.

#### Measures

##### Indifference

In this study, we examined recent feelings of indifference by asking participants to reflect upon the past two-week period. Therefore, all the items started with “Over the past two weeks. . .”. We measured *indifference* toward a romantic partner using the 8-item Subjective Interpersonal Indifference Scale (e.g., “Over the past two weeks, both the negative and positive feelings that I had toward my partner were rather weak”). Participants rated the extent to which the items applied to them on a 7-point scale (1 = *Did not apply to me at all*, 7 = *Completely applied to me*). As can be seen from the Cronbach’s alpha in Table 6, the scale once again showed good reliability, replicating Study 1. To further investigate the methodological soundness of the measure, we conducted a confirmatory factor analysis, which overall replicated the one-factor structure found in Study 1 (see Supplemental Material for details and results).

**Table 6.** Means, Standard Deviations, Bivariate Correlations, and Cronbach’s Alpha for Key Variables in Study 2.

Variable	*M* (*SD*)	1.	2.	3.	4.	5.	6.	7.	8.	9.	10.	11.	12.	13.	14.	15.
1. Indifference	2.50 (1.28)	.93														
2. Relationship satisfaction	5.20 (1.57)	−.73***	.96													
3. Commitment	6.08 (1.16)	−.67***	.73***	.89												
4. Thoughts of breaking up	2.22 (1.33)	.60***	−.74***	−.72***	.94											
5. Trust	5.56 (1.26)	−.61***	.77***	.71***	−.72***	.94										
6. Life satisfaction	4.66 (1.58)	−.50***	.70***	.49***	−.55***	.57***	.96									
7. Stress	1.09 (0.82)	.27***	−.34***	−.23***	.34***	−.38***	−.46***	.92								
8. Depression	0.74 (0.76)	.41***	−.48***	−.37***	.47***	−.50***	−.64***	.68***	.93							
9. Relational boredom	3.12 (1.34)	.80***	−.90***	−.73***	−.72***	−.75***	−.64***	.36***	.51***	.96						
10. Intimacy	4.81 (1.67)	−.72***	.84***	.67***	−.63***	.70***	.59***	−.32***	−.46***	−.87***	.92					
11. Desire for attractive alternatives	2.38 (1.66)	.32***	−.34***	−.40***	.32***	−.29***	−.20***	.11***	.15***	.35***	−.32***	.94				
12. Focus on work	4.54 (1.71)	−.04	.04	−.02	.06	.02	.03	−.02	−.10**	−.05	.06	.05	–			
13. Focus on hobbies	4.35 (1.49)	−.07*	.13***	.05	−.12***	.18***	.21***	−.27***	−.27***	−.15***	.14***	−.01	.07*	–		
14. Focus on other people	4.38 (1.45)	0.01	.00	−.02	.04	.00	.12***	−.04	−.12***	.02	−.03	.03	.09**	.13***	−	
15. General partner (d)evaluation	5.67 (1.29)	−.75***	.87***	.80***	−.71***	.76***	.60***	−.29***	−.42***	−.87***	.80***	−.34***	.04	.12***	.00	.95

*Note.* For each measure with more than one item, the Cronbach’s alpha is displayed on the diagonal.

* p < .05. **p < .01. ***p < .001.

##### Relationship Well-Being

As relationship outcomes, we measured *relationship satisfaction, commitment*, and *trust* using the same scales as in Study 1, and *thoughts of breaking up* using two items from Study 1 and an additional item to make it more suitable for the two-week timeframe that the study aimed to tap into (“Over the past two weeks, have you considered breaking up?”).

##### Personal Well-Being

As personal outcomes, we measured *life satisfaction, stress*, and *depression* using the same scales as in Study 1.

##### Mechanisms and Control Variable

As potential mechanisms, we measured *relational boredom* using the same scale as in Study 1, *intimacy* using three items from the Perceived Relationship Quality Component Inventory (Fletcher et al., 2000; e.g., “How intimate is your relationship?”), *desire for attractive alternatives* using three items (e.g., “Over the past two weeks, how much did you desire (physically and emotionally) someone other than your partner?”) adapted from Zoppolat et al. (2022), and *focus on work, focus on hobbies*, and *focus on other people* using one face-valid item each (“Over the past two weeks, how much did you focus on . . . Your work/Your hobbies/People other than your partner (such as friends, family, . . .)”, respectively), measured on a 7-point scale anchored at 1 = *Not at all*, 7 = *Completely/Extremely/Very much.* Finally, we measured the control variable *general partner (d)evaluation* using the same scale as in Study 1.

### Results

Table 6 shows the descriptive statistics, bivariate correlations, and reliability indices for the key variables in Study 2.

#### Associations of Indifference With Relationship and Personal Well-Being

The results of testing the total effects (see Table 5) showed that, controlling for general partner (d)evaluation, indifference was associated with all of the tested outcomes as hypothesized. Specifically, when it comes to relationship well-being, indifference was associated with lower relationship satisfaction, commitment, and trust, as well as higher thoughts of breaking up. Furthermore, in terms of personal well-being, indifference was associated with lower life satisfaction and higher depression. Indifference also had a significant positive link with stress.

#### Relational Boredom, Intimacy, and Attention to Other Domains as Mechanisms

Mediation analyses, controlling for general partner (d)evaluation, revealed significant indirect effects of indifference via higher relational boredom and lower intimacy on both relationship well-being indices (lower relationship satisfaction and trust, higher thoughts of breaking up) and personal well-being indices (lower life satisfaction, higher depression and stress). When testing attention to other domains outside of the relationship as mechanisms, only higher desire for attractive alternatives was a mediator of the associations between indifference and relationship outcomes (specifically, lower relationship satisfaction and commitment, and higher thoughts of breaking up), while focus on work, on hobbies, and on other people did not mediate these associations. Table 7 shows the full results of testing the indirect effects of indifference on relationship and personal outcomes via each of the mechanisms.

**Table 7.** Results of Testing Indirect Effects of Indifference on Relationship and Personal Outcomes Through Mechanisms, Controlling for General Partner (D)Evaluation in Study 2.

Outcome	Indirect effect of indifference via relational boredom			Indirect effect of indifference via intimacy			Indirect effect of indifference via desire for attractive alternatives			Indirect effect of indifference via focus on work			Indirect effect of indifference via focus on hobbies			Indirect effect of indifference via focus on other people		
	*ab*	*SE*	95% CI	*ab*	*SE*	95% CI	*ab*	*SE*	95% CI	*ab*	*SE*	95% CI	*ab*	*SE*	95% CI	*ab*	*SE*	95% CI
Relationship satisfaction	**−0.251**	**0.023**	**[−0.298, −0.207]**	**−0.118**	**0.018**	**[−0.157, −0.083]**	**−0.007**	**0.004**	**[−0.016, −0.001]**	−0.000	0.001	[−0.003, 0.002]	0.002	0.002	[−0.003, 0.007]	0.000	0.001	[−0.003, 0.003]
Commitment	−0.020	0.015	[−0.050, 0.009]	−0.010	0.009	[−0.029, 0.007]	**−0.018**	**0.007**	**[−0.034, −0.005]**	0.001	0.003	[−0.004, 0.007]	−0.002	0.002	[−0.007, 0.002]	−0.000	0.001	[−0.004, 0.002]
Thoughts of breaking up	**0.152**	**0.024**	**[0.106, 0.202]**	**0.041**	**0.016**	**[0.012, 0.074]**	**0.013**	**0.006**	**[0.003, 0.027]**	−0.002	0.005	[−0.013, 0.007]	−0.002	0.003	[−0.009, 0.003]	0.001	0.003	[−0.003, 0.007]
Trust	**−0.125**	**0.021**	**[−0.170, −0.087]**	**−0.062**	**0.015**	**[−0.093, −0.036]**	−0.005	0.004	[−0.015, 0.002]	0.000	0.001	[−0.002, 0.004]	0.004	0.005	[−0.005, 0.015]	0.000	0.001	[−0.003, 0.003]
Life satisfaction	**−0.231**	**0.032**	**[−0.295, −0.172]**	**−0.092**	**0.021**	**[−0.135, −0.054]**	–	–	–	–	–	–	–	–	–	–	–	–
Stress	**0.105**	**0.017**	**[0.073, 0.140]**	**0.039**	**0.011**	**[0.019, 0.062]**	–	–	–	–	–	–	–	–	–	–	–	–
Depression	**0.116**	**0.016**	**[0.084, 0.148]**	**0.048**	**0.010**	**[0.029, 0.068]**	–	–	–	–	–	–	–	–	–	–	–	–

*Note.* The significant indirect effects are indicated in bold. The dashes indicate that the respective mechanism was not tested as underlying the effects on a given outcome variable.

#### Findings From Analyses Using a Short Version of the Subjective Interpersonal Indifference Scale

We also tested the main hypothesized associations using three items (items 1–3 from Table 3) as a short version of the Subjective Interpersonal Indifference Scale, since these items were especially face-valid and had some of the highest factor loadings in Study 1. All conclusions from these analyses matched those from analyses using the full scale. Thus, these three items appear well-suited as a short measure of indifference toward a romantic partner (see Study 3).

### Discussion

The results of Study 2 showed that, as hypothesized, indifference toward a romantic partner was uniquely associated with relationship well-being (lower relationship satisfaction, commitment, and trust; higher thoughts of breaking up) and personal well-being (lower life satisfaction and higher depression) above and beyond general partner (d)evaluation. In this study, indifference was also associated with higher stress. Underlying these associations were higher relational boredom, higher desire for attractive alternatives, and lower intimacy. Finally, in Study 3, we aimed to replicate our main findings in a sample of romantic couples in the Netherlands. This study spanned 7 sessions, 6 months apart, over 3 years in total, which allowed us to test the hypotheses using both cross-sectional and, importantly, longitudinal analyses to examine associations over time.

## Study 3

### Method

#### Participants and Procedure

We recruited participants for the study in the Netherlands, primarily through a panel agency, with attention to diversity in their demographic characteristics (e.g., age, region, socioeconomic status). In determining the sample size, we considered recommendations in the research field (Finkel et al., 2015) and practical constraints. Participants were asked to fill out an online questionnaire every 6 months, over 7 sessions spanning 3 years in total. This study was not pre-registered.

#### Measures

##### Indifference

We measured *indifference* toward a romantic partner at all timepoints using a short 3-item version of the Subjective Interpersonal Indifference Scale. These three items are especially face-valid, had some of the highest factor loadings in Study 1, and replicated the findings from analyses where the full scale was used (see Study 2), therefore, they appeared highly adequate to measure the construct.

##### Relationship Well-Being

As relationship outcomes, at all timepoints, we measured *relationship satisfaction, commitment*, and *trust* using the same measures as in Studies 1 and 2, and *thoughts of breaking up* using a 3-item version of the scale from Study 1.

##### Personal Well-Being

As personal outcomes, at all timepoints, we measured *life satisfaction, stress*, and *depression* using the same scales as in Studies 1 and 2.

##### Mechanisms and Control Variable

As mechanisms, we measured *relational boredom* at all timepoints using a 9-item version of the scale used in Studies 1 and 2, and *intimacy* at Follow-ups 5 and 6 using the same scale as in Study 2. *Desire for attractive alternatives* was measured at all follow-up assessments using a single item (“Over the last six months, how often did you have thoughts of an (physically and emotionally) attractive other person?”; Zoppolat et al., 2022).^7^ As a control variable, we measured *general partner (d)evaluation* using the same scale as in Studies 1 and 2 at all timepoints.

#### Analytic Strategy

We tested our hypotheses using multilevel dyadic data analyses (Kenny et al., 2006) in three ways: using only variables measured at Intake (to test mechanisms that were only measured at Intake; see Supplemental Material for details), using the data from Intake and the six Follow-ups concurrently (i.e., in a cross-sectional design), and using the data from Intake and Follow-ups in a longitudinal design with time-lagged regression analyses (to test longitudinal associations). For the Intake-only analyses (which we report in the Supplemental Material), participants were nested within couples (two-level models), while in the analyses of multiple timepoints, participants were nested within couples and crossed with time points (crossed two-level models). All models included a random intercept and fixed slopes, and general partner (d)evaluation was included as a control variable. In testing for mediation effects, we once again used the joint-significance approach (MacKinnon et al., 2002) as in the previous studies. To construct a 95% confidence interval around the indirect effect estimate, we used the Monte Carlo method with 20,000 simulations (Selig & Preacher, 2008). For the time-lagged analyses, we tested models in which later relationship or personal outcomes were regressed onto earlier indifference, earlier general partner (d)evaluation, and earlier relationship or personal outcomes (for the total, un-mediated associations). To test for mediation in the time-lagged analyses, we ran models in which later relationship or personal outcomes were regressed onto earlier indifference, earlier general partner (d)evaluation, earlier relationship or personal outcomes, and earlier mechanisms. In other words, we tested mediation models in which the mediator was assessed at the same timepoint as the independent variable, and the dependent variable was assessed at a later timepoint (e.g., testing whether earlier indifference is associated with earlier relational boredom, which is in turn associated with later relationship satisfaction).^8^

### Results

Table 8 shows the descriptive statistics, bivariate correlations, and reliability indices for the key variables in Study 3.

**Table 8.** Means, Standard Deviations, Bivariate Correlations, and Cronbach’s Alpha for Key Variables in Study 3 Across Available Timepoints.

Variable	*M* (*SD*)	1.	2.	3.	4.	5.	6.	7.	8.	9.	10.	11.	12.
1. Indifference	2.04 (1.18)	.73											
2. Relationship satisfaction	5.99 (1.03)	−.58***	.93										
3. Commitment	6.39 (0.72)	−.48***	.61***	.68									
4. Thoughts of breaking up	2.26 (1.06)	.44***	−.57***	−.46***	.85								
5. Trust	5.93 (0.84)	−.47***	.66***	.48***	−.59***	.87							
6. Life satisfaction	5.61 (1.11)	−.36***	.57***	.33***	−.48***	.47***	.90						
7. Depression	0.40 (0.51)	.31***	−.30***	−.18***	.29***	−.25***	−.55***	.88					
8. Stress	0.74 (0.65)	.28***	−.30***	−.18***	.33***	−.27***	−.48***	.70***	.89				
9. Relational boredom	2.75 (0.94)	.51***	−.73***	−.43***	.45***	−.53***	−.49***	.30***	.32***	.87			
10. Intimacy	6.09 (0.98)	−.53***	.84***	.61***	−.53***	.66***	.53***	−.23***	−.26***	−.74***	.85		
11. Desire for attractive alternatives	2.64 (1.95)	.26***	−.31***	−.32***	.37***	−.25***	−.21***	.15***	.18***	.26***	−.30***	–	
12. General partner (d)evaluation	6.53 (0.66)	−.52***	.79***	.59***	−.44***	.61***	.41***	−.19***	−.22***	−.65***	.81***	−.22***	.92

*Note.* For each measure with more than one item, the Cronbach’s alpha is displayed on the diagonal.

*** p < .001.

#### Cross-Sectional Analyses

Table 5 shows the results of testing the total associations, and Table 9 shows the results of testing the indirect associations (through mechanisms) between indifference toward a romantic partner and relationship and personal well-being outcomes cross-sectionally across the seven study waves.

**Table 9.** Results of Testing the Indirect Effects of Indifference Through Mechanisms on Relationship and Personal Outcomes, Controlling for General Partner (D)Evaluation in Study 3.

Outcome	Design	Indirect effect of indifference via relational boredom		Indirect effect of indifference via intimacy		Indirect effect of indifference via desire for alternatives	
		*ab*	95% CI	*ab*	95% CI	*ab*	95% CI
Relationship satisfaction	Cross-sectional	**−0.045**	**[−0.055, −0.035]**	**−0.033**	**[−0.056, −0.012]**	**−0.007**	**[−0.013, −0.003]**
	Longitudinal	**−0.018**	**[−0.029, −0.009]**	**−0.019**	**[−0.041, −0.004]**	**−0.009**	**[−0.015, −0.003]**
Commitment	Cross-sectional	**−0.007**	**[−0.013, −0.002]**	**−0.010**	**[−0.021, −0.002]**	**−0.007**	**[−0.012, −0.003]**
	Longitudinal	−0.003	[−0.011, 0.004]	**−0.014**	**[−0.029, −0.003]**	**−0.007**	**[−0.012, −0.003]**
Thoughts of breaking up	Cross-sectional	**0.025**	**[0.017, 0.034]**	**0.021**	**[0.007, 0.039]**	**0.009**	**[0.004, 0.016]**
	Longitudinal	0.003	[−0.005, 0.012]	0.001	[−0.012, 0.014]	**0.010**	**[0.005, 0.017]**
Trust	Cross-sectional	**−0.017**	**[−0.023, −0.011]**	**−0.015**	**[−0.029, −0.005]**	**−0.005**	**[−0.009, −0.002]**
	Longitudinal	−0.005	[−0.011, 0.001]	**−0.012**	**[−0.026, −0.002]**	**−0.003**	**[−0.006, −0.000]**
Life satisfaction	Cross-sectional	**−0.037**	**[−0.048, −0.027]**	**−0.028**	**[−0.050, −0.009]**	–	–
	Longitudinal	**−0.010**	**[−0.020, −0.000]**	−0.005	[−0.021, 0.008]	–	–
Depression	Cross-sectional	**0.015**	**[0.010, 0.021]**	**0.008**	**[0.001, 0.018]**	–	–
	Longitudinal	0.005	[−0.001, 0.011]	−0.002	[−0.011, 0.007]	–	–
Stress	Cross-sectional	**0.018**	**[0.012, 0.025]**	0.006	[−0.002, 0.016]	–	–
	Longitudinal	0.007	[−0.000, 0.014]	0.002	[−0.009, 0.013]	–	–

*Note.* The significant indirect effects are indicated in bold. The dashes indicate that the respective mechanism was not tested as underlying the effects on a given outcome variable.

##### Associations of Indifference With Relationship and Personal Well-Being

In line with our predictions and replicating our previous studies, we found that, above and beyond general partner (d)evaluation, indifference toward a romantic partner was significantly associated with relationship well-being indices (lower relationship satisfaction, commitment, and trust, as well as higher thoughts of breaking up) and with personal well-being indices (lower life satisfaction and higher depression). The association between indifference and stress was once again significant and positive.

##### Relational Boredom, Intimacy, and Desire for Attractive Alternatives as Mechanisms

Mediation analyses showed that, when controlling general partner (d)evaluation, indifference was associated with relationship outcomes (lower relationship satisfaction, commitment, and trust, higher thoughts of breaking up) via higher relational boredom, via higher desire for attractive alternatives, and via lower intimacy. Furthermore, indifference was associated with personal outcomes (lower life satisfaction and higher depression) via higher relational boredom and via lower intimacy. Finally, indifference was associated with higher stress via higher relational boredom.

#### Longitudinal Analyses

Tables 5 (total effects) and 9 (indirect effects) also show the results of testing the longitudinal associations of indifference with relationship and personal well-being outcomes across the seven study waves.

##### Associations of Indifference With Later Relationship and Personal Well-Being

The results showed that earlier indifference was significantly associated with later relationship well-being (i.e., lower satisfaction, commitment, and trust, and higher thoughts of breaking up) and with later personal well-being (i.e., higher depression) when controlling for earlier levels of the outcome variable and earlier general partner (d)evaluation. The positive association with later stress was only marginally significant.

##### Relational Boredom, Intimacy, and Desire for Attractive Alternatives as Mechanisms

Mediation analyses showed that, when controlling for the earlier levels of the outcome variable and earlier general partner (d)evaluation, earlier indifference was associated with lower later relationship satisfaction and life satisfaction via higher earlier relational boredom. Furthermore, earlier indifference was associated with multiple later relationship well-being indices (lower relationship satisfaction, commitment, and trust, higher thoughts of breaking up) via higher earlier desire for attractive alternatives. Finally, earlier indifference was also associated with later relationship outcomes (lower relationship satisfaction, commitment, and trust) via lower earlier intimacy.

### Discussion

Study 3 provided further support for our hypotheses. Namely, across seven study waves, indifference toward a romantic partner was uniquely linked to both relationship well-being (lower relationship satisfaction, commitment, and trust; higher thoughts of breaking up) and personal well-being (lower life satisfaction; higher depression and stress) in a cross-sectional design. Underlying these associations were higher relational boredom, lower intimacy, and higher desire for attractive alternatives. Furthermore, when examining longitudinal associations across the seven study waves spanning 3 years in total, indifference toward a romantic partner uniquely predicted both relationship well-being (i.e., lower relationship satisfaction, commitment, and trust, and higher thoughts of breaking up) and personal well-being (i.e., higher depression) 6 months later. Our results also shed light on the underlying mechanisms of these longitudinal associations: Indifference was linked to higher relational boredom and desire for attractive alternatives, and lower intimacy, which predicted poorer well-being outcomes 6 months later. Importantly, all associations were unique to indifference, as we controlled for general (d)evaluation of the partner in all analyses.

## General Discussion

Although theoretical accounts of interpersonal evaluations (e.g., Righetti et al., 2026; Uchino et al., 2001) have recognized that people can have mostly positive, mostly negative, ambivalent (both positive and negative), and indifferent (neither positive nor negative) evaluations of their romantic partner, not all of these evaluation patterns have received equal empirical attention and conceptual development. For decades, mostly positive and mostly negative evaluative states in relationships have been the focus of considerable research efforts (for overviews, see Righetti et al., 2026; Ross et al., 2019), and research into ambivalence in relationships has been increasing only in recent years (e.g., Birmingham et al., 2019; Uchino et al., 2014; Zoppolat et al., 2023). However, empirical studies specifically addressing indifference toward a romantic partner have remained notably scarce, as have measurement tools specifically designed to assess such indifferent feelings. In the present work, we addressed this gap by investigating the associations of indifference toward a romantic partner with relationship and personal well-being, as well as their underlying mechanisms, both cross-sectionally and longitudinally, using the Subjective Interpersonal Indifference Scale. Across four studies, we find that experiencing indifference toward a romantic partner is linked to lower relationship well-being (as indicated by lower relationship satisfaction, commitment, trust, and higher thoughts of breaking up) and lower personal well-being (as indicated by lower life satisfaction and higher stress and depression), because it is associated with higher feelings of boredom in the relationship, higher desire for attractive alternatives, and lower intimacy. These associations were found when controlling for people’s general partner (d)evaluation, showing that feelings of indifference have unique associations with well-being and operate through specific mechanisms, above and beyond simply devaluing one’s partner.

Our findings integrate well with theoretical work on partner evaluations (e.g., Righetti et al., 2026; Ross et al., 2019; Uchino et al., 2001), where indifference in a romantic relationship has been proposed to be a distressing and non-normative experience, which likely provokes increasing disinterest and distancing from the partner, and has negative well-being consequences. Indeed, we show that indifference is associated with lower relationship well-being (i.e., lower relationship satisfaction, commitment, and trust, and more thoughts of breaking up) and personal well-being (i.e., lower life satisfaction and higher depression). These associations were found both when indifference and well-being outcomes were measured at the same timepoint (i.e., cross-sectionally) and when earlier indifference was predicting well-being outcomes 6 months later, controlling for earlier well-being (i.e., longitudinally). Particularly intriguing are the findings regarding the association between indifference and stress. Namely, we found that indifference was linked to higher stress cross-sectionally in Studies 2 and 3 (and in the Pilot Study reported in the Supplemental Material), but not in Study 1. Furthermore, the longitudinal association with stress was not significant in Study 3. Taken together, these findings indicate that even though indifference is generally a relatively passive and de-activating state (e.g., Schneider et al., 2016) and might not be stressful when directed at most attitude objects, it might be stressful when experienced toward one’s romantic partner. One possible explanation for this is that experiencing indifference toward a partner conflicts with personal, social, or cultural expectations regarding how people should feel in romantic relationships. Nevertheless, because the association between indifference and stress was not consistently observed, these interpretations should be viewed with caution, and further research is needed to clarify this link and identify its potential boundary conditions. We also identified several key mechanisms through which indifference operates in romantic relationships. Given that indifference is a low-arousal state (Schneider et al., 2016) that may signal that the partner is unstimulating and unlikely to fulfill one’s needs (Righetti et al., 2026), is appraised as unimportant (Cohen-Chen et al., 2022; van Kleef, 2016), and is therefore not particularly worth engaging with (Gasper et al., 2019), we expected that it would be linked to several specific psychological mechanisms that reflect these processes. In line with our hypotheses, we found that feelings of indifference toward a romantic partner were associated with higher feelings of relational boredom, higher desire for attractive alternatives, and lower intimacy in the relationship, which were in turn linked to poorer relationship and personal well-being outcomes. These associations also held when examined over time, showing that indifference was associated with higher relational boredom, higher desire for attractive alternatives, and lower intimacy, which were in turn associated with poorer well-being outcomes 6 months later. Interestingly, although we did not originally hypothesize a causal chain of processes, these mechanisms could also be linked to each other. For example, indifference could be linked to higher feelings of relational boredom which, *in turn*, could motivate desire for attractive alternatives or engaging in other activities that might provide fulfillment (e.g., Westgate & Wilson, 2018). These processes could subsequently be associated with lower relationship well-being. In addition, these mechanisms could have implications for other relationship processes that also influence the future of the relationship. For example, there is some evidence that while people believe that they *should* change the activities that they engage in with their partner in order to alleviate relational boredom (Harasymchuk et al., 2017), experiencing relational boredom is linked to *less* actual engagement in exciting activities with one’s partner (Harasymchuk et al., 2022). This could be one way through which indifference perseveres and leaves a lasting mark on relationships: Indifferent individuals experience boredom and a lack of intimacy with their partner, and although they might feel that they *should* engage in relationship repair, they remain passive – in turn potentially reinforcing their indifferent state and relationship deterioration. At any rate, taken together, our findings show that experiencing indifference toward a romantic partner might be very impactful both in terms of its more immediate consequences and in the long run.

Investigations of these processes were made possible by the development of the Subjective Interpersonal Indifference Scale, which showed satisfactory validity and reliability (.73 > α > .93) in measuring indifference toward a romantic partner across our studies. Importantly, we note that the scale items could easily be rephrased to measure indifference toward people other than a romantic partner as well. Indeed, theoretical accounts of interpersonal evaluations (e.g., Righetti et al., 2026; Uchino et al., 2001) have argued that indifference can be experienced toward various members of one’s social network – next to romantic partners, these can be friends, family members, co-workers, acquaintances, etc. With the Subjective Interpersonal Indifference Scale as a specific measurement tool for assessing the extent to which indifference is felt toward another person, we hope to stimulate further investigations of interpersonal indifference to systematically document its effects across people’s social networks. Following the statistical approach employed in the present work, we advise controlling for related variables such as general evaluations of the target person when examining indifference, to ensure that its unique effects are detected. Indeed, indifference is likely to have specific negative effects on well-being when it is experienced toward close others and in relationships where high emotional engagement is expected (see also Cohen-Chen et al., 2022). For instance, we expect that indifference would be distressing and detrimental when experienced toward close friends and immediate family members. In contrast, it might not be (as) upsetting or impactful for well-being to experience indifference toward more distal others, especially those who do not hold much influence over people’s outcomes – such as certain coworkers, neighbors, acquaintances, or strangers. These considerations offer important insights into the nature of indifference in social contexts. Namely, while indifference should not be inherently aversive (i.e., negative evaluations/feelings are low, therefore, the experiencer should not find this state unpleasant or distressing), it appears that it can carry substantial negative meaning when it is experienced in certain relational or social contexts. This may be especially true when indifference is perceived as non-normative, thereby conflicting with personal or cultural expectations (Cohen-Chen et al, 2022). Nevertheless, the well-being consequences, underlying mechanisms, and even potential beneficial functions of indifference toward other people remain to be examined by future studies, as it is also possible that people might use indifference functionally. For example, indifference might be employed as an emotional “shield”, in an attempt to disengage emotionally, protect one’s vulnerability, or avoid greater emotional turmoil (see e.g., Gross, 2002) in contexts where experiencing strong positive, negative, or ambivalent feelings would keep one engaged with a potentially hurtful situation (e.g., unrequited love or friendship, ex-partners or ex-friends, frustrating coworkers).

Other important avenues for future research on indifference within the domain of romantic relationships also remain. It is particularly relevant for future studies to examine indifference in couples who are in distress and/or considering relationship dissolution, as greater variance in indifference would likely be detected in such samples, and the findings from such studies would have especially important clinical implications. In this context, measuring indifference could also inform the study of related constructs that have been relevant to the treatment of distressed couples (e.g., Abbasi & Alghamdi, 2017). Namely, measuring partner evaluations such as indifference can uncover the underlying evaluative structure behind processes like romantic disengagement and disaffection, which would be informative for disentangling attitudes and feelings from behaviors, and subsequently adjusting interventions. Furthermore, as romantic relationships are marked by high interdependence (Kelley & Thibaut, 1978), whereby the feelings and actions of one partner greatly affect the other partner’s outcomes, it is likely that people are not only affected by their own feelings of indifference toward their partner, but also by their *partner’s* indifference toward them. Indeed, recent research has shown that people predominantly respond negatively to indifference/neutrality displayed by others, such as in conflict negotiations (Cohen-Chen et al., 2022) or among friends (Shaw et al., 2017). Future research could examine the well-being consequences of having such a romantic partner who is experiencing indifference toward the individual, and whether the individual in turn responds to this by engaging (e.g., trying to improve the relationship; see also Dai et al., 2014) or disengaging from the partner (e.g., increasing their own indifference and behavioral avoidance; see also Cohen-Chen et al., 2022; Shaw et al., 2017). Future studies could also investigate the possibility that experiencing indifference toward a current romantic partner may not be equally impactful for everyone’s well-being. For example, people with high levels of attachment avoidance might be less distressed by such indifference, as they are more prone to indifferent reactions to their partners (Carnelley et al., 2007) and therefore may consider indifference to be less of a non-normative and alarming experience in relationships than people with a more secure attachment style. Finally, future work could examine how and when feelings of indifference arise in romantic relationships. This could be a gradual process where previously mostly positive, ambivalent, or mostly negative partner evaluations fade away over the course of a relationship as individuals emotionally withdraw from it (Righetti et al., 2026). Alternatively, in some cases, the responsible process may not be tied to the relationship at all – for instance, individuals suffering from depression (e.g., Proudfit et al., 2015) or with a history of childhood trauma (e.g., Wang et al., 2022) may be prone to general indifference or emotional disengagement, which might spill over into indifferent feelings toward a romantic partner as well.

### Strengths and Limitations

We note several limitations of this work. Many of our studies examined the associations between indifference and outcomes cross-sectionally; thus, causal interpretations of the results must be taken with caution. However, the longitudinal study shed light on the temporal dynamics of the effects, by showing that earlier indifference predicted later relationship and personal outcomes when controlling for earlier outcomes. Although a laboratory manipulation could have provided additional insights into causality, such an approach might be particularly challenging in the context of romantic relationships. In fact, evaluations in such relationships are deeply rooted in a long history between the partners, making them difficult to manipulate in a short experimental setup. Thus, measuring indifference remains the most viable and ecologically valid approach to testing its effects. Furthermore, our studies only examined self-reported outcomes as we were interested in psychological indicators of well-being, which are best reported by the individuals themselves. However, it is important for future research to also investigate how more objective indicators of relationship and personal well-being (e.g., break-up decisions, physiological indexes related to health) are affected by indifference. Finally, we note that our studies sampled from relatively WEIRD (Western, Educated, Industrialized, Rich, and Democratic; Henrich et al., 2010) countries, while there may be cultural differences in the experience and consequences of indifference in romantic relationships. For example, there is some research showing that romance is thought to be particularly important in relationships in Western cultures (e.g., Medora et al., 2002), although there is also work showing no cross-cultural differences in experiences of passionate love (for a review, see Hatfield & Rapson, 2006). Therefore, while we demonstrate that indifference toward a romantic partner is seen as non-normative and linked to negative outcomes in Western cultural contexts, it remains an open empirical question whether experiencing indifference toward a romantic partner is equally distressing in other cultural contexts.

We also highlight several key strengths of this work. We investigated an evaluative state that has been almost completely neglected in previous empirical research by bridging concepts and theories from the literature on attitudes/evaluations and from the field of romantic relationships. To facilitate future investigations of this important construct, we developed a new measure of interpersonal indifference, which showed satisfactory psychometric properties. In terms of methodology, we emphasize that our samples spanned three countries, including both couples and individuals who are in romantic relationships, and were relatively diverse in terms of demographic characteristics (gender balance, age range, relationship duration). We replicated our findings across four studies, using both cross-sectional and longitudinal analyses, and controlling for general partner (d)evaluation to rebut a potential alternative explanation (i.e., that the associations arise due to partner devaluation rather than indifference).

## Conclusion

In contrast to the strong positive, and occasional negative, evaluations that people expect to experience in romantic relationships, some individuals may instead experience indifference toward their romantic partner. Our findings reveal that this state – characterized by simultaneously low positive and negative evaluations – can be both distressing and harmful. Across four studies, we demonstrate that indifference toward a romantic partner is linked to higher relational boredom, higher desire for alternative partners, and lower intimacy, which are in turn associated with poorer relationship and personal well-being. We encourage researchers to explore this under-investigated state further, as it represents the evaluative reality for some couples, with links to adverse and potentially long-lasting well-being outcomes.

## Supplemental Material

sj-docx-1-psp-10.1177_01461672251410278 – Supplemental material for Just Not That Into You: Experiences of Indifference Toward a Romantic Partner Supplemental material, sj-docx-1-psp-10.1177_01461672251410278 for Just Not That Into You: Experiences of Indifference Toward a Romantic Partner by Mirna Đurić, Francesca Righetti, Giulia Zoppolat and Iris K. Schneider in Personality and Social Psychology Bulletin

## Data Availability

Data for the Pilot Study, Study 1, and Study 2 are available on the project’s OSF page (https://osf.io/mfuz2/overview?view_only=f2aa6fef9bf344db803932e6ab33eba7), while data for Study 3 are not publicly available due to ethical regulations (phrasing of the informed consent form and the sensitivity of dyadic data).

## Notes

1. We focus on subjective indifference as we anticipate that it is more consequential for the experiencer than *objective* indifference, which could be assessed through mathematical formulas that have been commonly applied in research on ambivalence, but that only allow assessment of the underlying structure of the evaluative state, rather than an individual’s experience of it.

2. Three of our studies also examined associations of indifference with relationship security. However, as this construct has considerable conceptual overlap with trust, we report these results in the Supplemental Material.

3. We also tested the significant mechanisms as parallel mediators in Studies 2 and 3 to assess their relative contributions. These results are reported in the Supplemental Material and have overall produced similar conclusions to the analyses where the mechanisms were tested in separate models (which we report in the main text, as they were pre-registered and as we did not have any a priori expectations about the relative contributions of the mechanisms).

4. Although the results of the Pilot Study supported our hypotheses, we report it in the Supplemental Material since the sample had specific characteristics (individuals who have never experienced romantic jealousy and/or are in an open relationship) and the indifference measure had not been fully developed yet.

5. Aiming at increased transparency of reporting, we note that although we present Study 3 as the final study in the paper (due to its complexity), we analyzed parts of the data from this study alongside the Pilot Study for an initial test of our hypotheses. Given that we used parts of Study 3 in a more exploratory way, we did not pre-register its analyses.

6. We note that we pre-registered relational boredom as a control variable in the analyses testing criterion validity. However, as relational boredom is identified as a mechanism, we report the mediation analyses instead in the main text, with the pre-registered analyses controlling for boredom available in the Supplemental Material.

7. Desire for an attractive alternative was also measured at Intake using two different measures; the measurement details and results of testing this mechanism at Study 3 Intake are described in the Supplemental Material. Several different measures were also included at Intake and some follow-up sessions to test attention to other domains – family, friends, work, hobbies, other goals – as mechanisms; measurement details and results are also reported in the Supplemental Material.

8. Given that we did not have a priori expectations regarding the temporal nature of the indirect effects, we also tested mediation models in which the mediator and the dependent variable were assessed at a later timepoint than the independent variable (e.g., testing whether earlier indifference is associated with later intimacy, which is in turn associated with later relationship satisfaction). We found that indifference was associated with higher later relational boredom, which was in turn linked to lower later relationship and personal well-being. The indirect effects of indifference on well-being via later intimacy and later desire for attractive alternatives were not significant. The statistics are reported in the Supplemental Material.
