# Supplementary material for: Just Not That Into You: Experiences of Indifference Toward a Romantic Partner
Source: Pers Soc Psychol Bull. 2026 Jan 8;52(12):4243–64. doi: 10.1177/01461672251410278 (PMC13736278; doi:10.1177/01461672251410278)
Supplement: Supplementary file 1 [file sj-docx-1-psp-10.1177_01461672251410278.docx]

**Supplementary Materials**

Just Not That Into You: Experiences of Indifference Toward a Romantic Partner

**Table of Contents**

Supplement A: Pilot Study

Supplement B: Additional Results Study 1

Supplement C: Additional Results Study 2

Supplement D: Additional Results Study 3

**Supplement A**

**Pilot Study**

We report the Pilot Study in this Supplement, where we first tested our hypotheses in a sample of individuals involved in a romantic relationship recruited on Prolific. We examined the associations between indifference and relationship and personal well-being, and started exploring one of the proposed mechanisms: relational boredom. Furthermore, this Supplement contains analyses of relationship security as a relationship well-being outcome and a list of measures included in the Pilot Study but not used in the present analyses.

**Method**

***Participants***

Participants were recruited as part of another study about romantic jealousy (see: <https://osf.io/nyd2b/overview?view_only=ff13ad0b71664d23aa2d50f4a6b1d040> and Đurić, Righetti, Zoppolat, Lohmer, & Schneider, 2025). Specifically, 1000 participants who were in a romantic relationship for at least four months, resided in the US or the UK, and did not fail two or more of our attention checks were initially included in the sample. Seven of these participants who reported that we should discard their data because they gave some random responses and/or responses that were not completely honest, and one participant who indicated a relationship duration below four months in the demographic questions, were excluded from further analyses. Upon accessing the survey, participants were asked whether they were currently in an open relationship with their partner and whether they had ever experienced romantic jealousy in their current relationship. Participants who were currently *not* in an open relationship and indicated that they *had* experienced romantic jealousy in their current relationship continued with the study about romantic jealousy, while the remaining participants were redirected to this study instead (i.e., these were participants who were in an open relationship, preferred not to answer whether they are in an open relationship, or were not in an open relationship but had no experiences of romantic jealousy). This yielded a final sample of 559 participants for the current study (48.5% women, 50.6% men, 0.5% selected option “other” for gender, 0.4% preferred not to answer; *M*_age_ = 44.5, *SD*_age_ = 13.4, age range 19-81; 97.5% residing in the UK, 2.1% residing in the US, 0.4% preferred not to answer; *M*_relationship duration_ = 192.1 months/16.0 years, *SD*_relationship duration_ = 151.7 months/12.6 years, relationship duration range 5-672 months/56.0 years; 90.2% living together with partner; 61.7% married; 4.5% in an open relationship, 95.0% not in an open relationship, 0.5% preferred not to answer). Sensitivity analyses using G*Power v. 3.1.9.7 (Faul et al., 2009) showed that a replication study would have 80% power to detect small effect sizes (*f^2^* = 0.014) in a multiple regression analysis with two predictors (indifference and one control variable).

***Measures***

All measures were rated on a 7-point scale (1 = Not at all/Do not agree at all; 7 = Completely/Completely agree), unless noted otherwise.

**Indifference.** We measured *indifference* toward a romantic partner using three face-valid items (“I do not feel strong emotions toward my partner (either positive or negative)”, “Sometimes I feel a bit numb toward my partner”, “Sometimes I feel a bit indifferent toward my partner”).

**Relationship Well-Being.** As indexes of relationship well-being, we measured *relationship satisfaction, commitment,* and *thoughts of breaking up* using the same measures as in Study 1*,* and *trust* using a face-valid single item (“I trust my partner”)*.*

**Personal Well-Being.** As indexes of personal well-being, we measured *life satisfaction* using the same measure as in Study 1, and *depression* and *stress* using three items from the respective subscales of the Depression, Anxiety, and Stress Scale (DASS-21; Lovibond & Lovibond, 1995; e.g., “I felt down-hearted and blue” and “I found it difficult to relax”, respectively) which were rated on a 4-point scale (0 = Did not apply to me at all, 3 = Applied to me very much or most of the time)*.*

**Mechanism and Control Variable.** We measured *relational boredom* using three items adapted from the scale used in Study 1, and participants’ *general partner (d)evaluation* using a 3-item version of the scale used in Study 1.

***Procedure***

After being redirected to the current study, participants filled out the online questionnaire that contained the described measures and demographic questions. Upon survey completion, participants were debriefed and paid 1 GBP for their participation, in line with Prolific guidelines. This study was not pre-registered.

***Analytic Strategy***

First, we conducted multiple linear regression analyses with each relationship/personal well-being outcome regressed on indifference (our key independent variable) and general partner (d)evaluation (control variable). To test the mediation hypotheses, we used the same approach as described for Study 1 in the main text.

**Results**

Table A1 shows the descriptive statistics, bivariate correlations, and reliability indices for the key variables in the Pilot Study.

**Table A1**

*Means, Standard Deviations, Bivariate Correlations, and Cronbach’s Alpha for Key Variables in the Pilot Study*

| **Variable** | ***M***  **(*SD*)** | **1.** | **2.** | **3.** | **4.** | **5.** | **6.** | **7.** | **8.** | **9.** | **10.** |
| --- | --- | --- | --- | --- | --- | --- | --- | --- | --- | --- | --- |
| **1. Indifference** | 2.21 (1.40) | .89 |  |  |  |  |  |  |  |  |  |
| **2. Relationship satisfaction** | 5.58 (1.35) | -.74*** | .96 |  |  |  |  |  |  |  |  |
| **3. Commitment** | 6.29 (0.99) | -.64*** | .76*** | .86 |  |  |  |  |  |  |  |
| **4. Thoughts of breaking up** | 2.03 (1.08) | .63*** | -.69*** | -.67*** | .90 |  |  |  |  |  |  |
| **5. Trust** | 6.52 (0.91) | -.55*** | .64*** | .63*** | -.60*** | – |  |  |  |  |  |
| **6. Life satisfaction** | 5.11 (1.33) | -.43*** | .64*** | .47*** | -.46*** | .41*** | .95 |  |  |  |  |
| **7. Stress** | 0.93 (0.82) | .26*** | -.26*** | -.23*** | .33*** | -.25*** | -.43*** | .87 |  |  |  |
| **8. Depression** | 0.85 (0.83) | .36*** | -.38*** | -.30*** | .40*** | -.28*** | -.59*** | .76*** | .85 |  |  |
| **9. Relational boredom** | 2.15 (1.40) | .70*** | -.80*** | -.67*** | .64*** | -.57*** | -.49*** | .24*** | .36*** | .94 |  |
| **10. General partner (d)evaluation** | 6.39 (0.97) | -.71*** | .82*** | .82*** | -.67*** | .65*** | .49*** | -.20*** | -.30*** | -.75*** | .95 |

*Note.* Cronbach’s alpha for each scale with more than one item is shown on the diagonal.

*** *p* < .001

The results of testing the total effects and the indirect effects (through mechanisms) of indifference on relationship and personal outcomes are shown in Table A2.

***Associations of Indifference With Later Relationship and Personal Well-Being***

As hypothesized, above and beyond general partner (d)evaluation, indifference was linked to relationship well-being (lower relationship satisfaction, commitment, and trust; higher thoughts of breaking up) and personal well-being (lower life satisfaction and higher depression). We also found that indifference was associated with higher stress.

***Relational Boredom as a Mechanism***

The mediation models also supported our hypotheses. Namely, above and beyond general partner (d)evaluation, indifference was linked to higher relational boredom, which was, in turn, associated with relationship well-being (lower relationship satisfaction and higher thoughts of breaking up) and personal well-being (lower life satisfaction and higher depression).

**Table A2**

*Results of Testing Total and Indirect Effects of Indifference Through Relational Boredom on Relationship and Personal Outcomes, Controlling for General Partner (D)Evaluation in the Pilot Study*

| **Outcome** | **Total effect of indifference** | | | | **Indirect effect of indifference via relational boredom** | | |
| --- | --- | --- | --- | --- | --- | --- | --- |
|  | *b* | *SE* | *t* | *p* | *ab* | *SE* | 95% CI |
| **Relationship satisfaction** | -0.31 | 0.03 | -10.46 | < .001 | -0.113 | 0.021 | [-0.157, -0.075] |
| **Commitment** | -0.09 | 0.02 | -3.83 | < .001 | -0.021 | 0.012 | [-0.047, 0.003] |
| **Thoughts of breaking up** | 0.24 | 0.03 | 7.28 | < .001 | 0.056 | 0.017 | [0.025, 0.092] |
| **Trust** | -0.12 | 0.03 | -4.17 | < .001 | -0.033 | 0.020 | [-0.076, 0.004] |
| **Life satisfaction** | -0.15 | 0.05 | -3.02 | .003 | -0.087 | 0.023 | [-0.135, -0.045] |
| **Stress** | 0.13 | 0.03 | 3.91 | < .001 | 0.029 | 0.016 | [-0.001, 0.061] |
| **Depression** | 0.18 | 0.03 | 5.35 | < .001 | 0.045 | 0.017 | [0.016, 0.081] |

**Discussion**

The results of the Pilot Study provided preliminary support for our hypotheses. Feelings of indifference toward a romantic partner were uniquely associated with relationship well-being (lower relationship satisfaction, commitment, and trust, as well as more thoughts of breaking up) and personal well-being (lower life satisfaction and higher depression), above and beyond general partner (d)evaluation. We also found that indifference was associated with higher stress. Underlying these associations was higher relational boredom. Although the Pilot Study provided considerable support for our hypotheses regarding the associations between indifference toward one’s partner and lower relationship and personal well-being, it gathered data from a specific sample of people who did not experience jealousy in their relationship and/or were in an open relationship, and measured indifference using three items for which there was no information about their validity. To address these shortcomings, in Study 1 we developed and validated a new scale to measure interpersonal indifference and sought to replicate the findings in a study specifically designed and preregistered to test the hypotheses.

**Relationship Security as a Relationship Well-Being Outcome**

As noted in the main text, we also tested relationship security as a relationship well-being outcome. We measured relationship security using three face-valid items (e.g., “I feel safe and secure of the bond I have with my partner”; Đurić, Righetti, Zoppolat, Solleiro Saura, & Schneider, 2025), rated on a 7-point scale (1 = Do not agree at all; 7 = Completely agree). The mean, standard deviation, Cronbach’s alpha, and bivariate correlations of relationship security with other key variables in the Pilot Study are shown in Table A3.

**Table A3**

*Means, Standard Deviations, Bivariate Correlations, and Cronbach’s Alpha for Key Variables in the Pilot Study*

| **Variable** | ***M***  **(*SD*)** | **1.** | **2.** | **3.** | **4.** | **5.** | **6.** | **7.** | **8.** | **9.** | **10.** | **11.** |
| --- | --- | --- | --- | --- | --- | --- | --- | --- | --- | --- | --- | --- |
| **1. Indifference** | 2.21 (1.40) | .89 |  |  |  |  |  |  |  |  |  |  |
| **2. Relationship satisfaction** | 5.58 (1.35) | -.74*** | .96 |  |  |  |  |  |  |  |  |  |
| **3. Commitment** | 6.29 (0.99) | -.64*** | .76*** | .86 |  |  |  |  |  |  |  |  |
| **4. Thoughts of breaking up** | 2.03 (1.08) | .63*** | -.69*** | -.67*** | .90 |  |  |  |  |  |  |  |
| **5. Relationship security** | 5.97  (1.29) | -.68*** | .79*** | .77*** | -.71*** | .88 |  |  |  |  |  |  |
| **6. Trust** | 6.52 (0.91) | -.55*** | .64*** | .63*** | -.60*** | .66*** | – |  |  |  |  |  |
| **7. Life satisfaction** | 5.11 (1.33) | -.43*** | .64*** | .47*** | -.46*** | .48*** | .41*** | .95 |  |  |  |  |
| **8. Stress** | 0.93 (0.82) | .26*** | -.26*** | -.23*** | .33*** | -.26*** | -.25*** | -.43*** | .87 |  |  |  |
| **9. Depression** | 0.85 (0.83) | .36*** | -.38*** | -.30*** | .40*** | -.32*** | -.28*** | -.59*** | .76*** | .85 |  |  |
| **10. Relational boredom** | 2.15 (1.40) | .70*** | -.80*** | -.67*** | .64*** | -.68*** | -.57*** | -.49*** | .24*** | .36*** | .94 |  |
| **11. General partner (d)evaluation** | 6.39 (0.97) | -.71*** | .82*** | .82*** | -.67*** | .79*** | .65*** | .49*** | -.20*** | -.30*** | -.75*** | .95 |

*Note.* Cronbach’s alpha for each scale with more than one item is shown on the diagonal.

*** *p* < .001

We applied the same analytic approach as described for the other relationship and personal outcomes. The results showed that, controlling for general partner (d)evaluation, indifference was significantly negatively associated with relationship security (*b* = -0.22; *SE* = 0.03; *t* = -6.92; *p* < .001). Indifference was also significantly associated with lower relationship security via higher relational boredom (indirect effect *ab* = -0.039; *SE* = 0.02; 95% CI [-0.073, -0.007]).

**Other Measures Included in the Study**

The following measures were included in the study but not used in the present analyses because they were assessed to answer a different research question: perceived partner indifference, perceived partner responsiveness, perceived partner’s general partner (d)evaluation, perceived partner relational boredom, demographic variables.

**Supplement B**

**Additional Results Study 1**

This Supplement contains analyses of psychometric properties of the Subjective Interpersonal Indifference Scale, additional factor analyses (indifference, relational boredom, general partner (d)evaluation, disengagement, disaffection), sensitivity analyses, analyses of relationship security as a relationship well-being outcome, and tests of criterion validity when controlling for relational boredom in Study 1.

**Analyses of Psychometric Properties of the Subjective Interpersonal Indifference Scale**

***Scale Construction***

To examine the properties of the indifference scale, we started by conducting an exploratory factor analysis with principal axis factoring. Given the variety of methods that have been recommended in the literature for determining the number of factors to extract (see e.g., Boateng et al., 2018; Watkins, 2018), we took into account multiple criteria: eigenvalues, the scree plot, the variance explained by a factor, parallel analysis and the minimum average partial (MAP) test as additional analytic procedures, and theoretical considerations. We pre-registered our plans to retain, at most, eight best-performing items in the scale to keep it relatively brief. We also pre-registered our requirements that these items would have a factor loading of at least .40 and show positive corrected item-total correlations with the total scale score of at least .30.

Overall, exploratory factor analyses with the interpersonal indifference items indicated a one-factor solution as the most plausible (see below for scree plot, parallel analysis and MAP test results, and considerations of multiple-factor solutions). This factor explained 56% of the variance in responses and had an eigenvalue of 7.85. The factor loadings found in the one-factor solution, means and standard deviations, and corrected item-total correlations for each of the items can be seen in Table 3 in the main text. All items satisfied our criteria of having a factor loading of at least .40 and a positive corrected item-total correlation of at least .30. Therefore, to keep the final Subjective Interpersonal Indifference Scale relatively brief, we retained the eight best-performing items based on factor loadings, which are indicated in bold text in Table 3 in the main text. The Cronbach’s alpha of .92 indicated that the measure had good reliability (see below for examinations of Cronbach’s alpha if items are deleted). The total scale score was formed by calculating an average across the individual items.

***Scree Plot, Parallel Analysis, and MAP Test***

Figure B1 shows the scree plot (Cattell, 1966) with the eigenvalues of the factors that could be extracted from the initial item pool. We interpreted this plot as indicating that one-, two-, and three-factor solutions might be considered.

**Figure B1**

*Scree Plot for the Subjective Interpersonal Indifference Scale Item Pool in Study 1*

To further examine plausible factor solutions, we conducted a parallel analysis (Horn, 1965) using the psych package (Revelle, 2024) and the minimum average partials (MAP) test (Velicer, 1976; Velicer et al., 2000) using the EFA.dimensions package (O’Connor, 2024) in R (v. 4.4.0; R Core Team, 2024). The R code for these analyses is available in the project OSF folder (<https://osf.io/mfuz2/overview?view_only=f2aa6fef9bf344db803932e6ab33eba7>). The output of the parallel analysis indicated that the number of factors is three and the number of components is one, with the accompanying scree plot displayed in Figure B2. The MAP test indicated that the number of components according to both the original (Velicer, 1976) and revised (Velicer et al., 2000) MAP test is two. Based on these considerations, we proceeded by examining a one-, two-, and three-factor solution for the scale.

**Figure B2**

*Parallel Analysis Scree Plot for the Subjective Interpersonal Indifference Scale Item Pool in Study 1*

***Two-Factor and Three-Factor Solutions***

In addition to the one-factor solution reported above, we considered a two-factor and a three-factor solution for the Subjective Interpersonal Indifference Scale. The second factor explained 8% of the variance in responses and had an eigenvalue of 1.14, while the third factor explained less than 5% of the variance in responses and had an eigenvalue of 0.66.

**Two-Factor Solution.** Two factors were extracted with promax rotation (kappa = 4). The two factors were correlated at *r* = .76. The pattern matrix and structure matrix are reported in Table B1.

**Table B1**

*Pattern Matrix and Structure Matrix for the Two-Factor Solution in Study 1*

| **Item** | **Pattern Matrix** | | **Structure Matrix** | |
| --- | --- | --- | --- | --- |
|  | **Factor 1** | **Factor 2** | **Factor 1** | **Factor 2** |
| 1. I do not feel strong emotions toward my partner at all (neither positive nor negative). | .506 | .333 | .757 | .715 |
| 2. I feel a bit numb toward my partner. | .339 | .480 | .701 | .736 |
| 3. I feel a bit indifferent toward my partner. | .442 | .425 | .763 | .759 |
| 4. When I think about my partner, my thoughts are neither very negative nor very positive. | .723 | .027 | .743 | .572 |
| 5. I do not feel much disappointment nor much excitement toward my partner. | .772 | -.068 | .721 | .515 |
| 6. My partner is not a source of joy for me but they are also not a frustration. | .758 | -.096 | .686 | .476 |
| 7. I find it difficult to feel strong emotions toward my partner. | .402 | .437 | .732 | .741 |
| 8. I feel intense emotions toward my partner. * | -.171 | .973 | .563 | .843 |
| 9. When I think about my partner, I feel powerful emotions. * | -.059 | .879 | .604 | .834 |
| 10. I have strong feelings toward my partner. * | .014 | .833 | .643 | .844 |
| 11. Both the negative and positive feelings that I have toward my partner are rather weak. | .672 | .080 | .732 | .587 |
| 12. I do not particularly highly value my partner but I also do not look down on them. | .712 | -.009 | .705 | .528 |
| 13. I do not have an extremely positive or an extremely negative opinion of my partner. | .703 | -.016 | .690 | .514 |
| 14. I feel neutral toward my partner. | .503 | .254 | .695 | .634 |

*Note.* Items with an asterisk (*) are reverse-scored.

**Three-Factor Solution.** Three factors were extracted with promax rotation (kappa = 4). The first and the second factor were correlated at *r* = .71, the first and the third factor at *r* = .78, and the second and the third factor at *r* = .76. The pattern matrix and structure matrix are reported in Table B2.

**Table B2**

*Pattern Matrix and Structure Matrix for the Three-Factor Solution in Study 1*

| **Item** | **Pattern Matrix** | | | **Structure Matrix** | | |
| --- | --- | --- | --- | --- | --- | --- |
|  | **Factor 1** | **Factor 2** | **Factor 3** | **Factor 1** | **Factor 2** | **Factor 3** |
| 1. I do not feel strong emotions toward my partner at all (neither positive nor negative). | .439 | .218 | .200 | .749 | .679 | .707 |
| 2. I feel a bit numb toward my partner. | -.082 | .008 | .942 | .658 | .663 | .884 |
| 3. I feel a bit indifferent toward my partner. | .100 | .006 | .800 | .728 | .683 | .883 |
| 4. When I think about my partner, my thoughts are neither very negative nor very positive. | .749 | .057 | -.047 | .752 | .550 | .580 |
| 5. I do not feel much disappointment nor much excitement toward my partner. | .765 | -.049 | -.006 | .726 | .487 | .554 |
| 6. My partner is not a source of joy for me but they are also not a frustration. | .754 | -.070 | -.017 | .691 | .449 | .518 |
| 7. I find it difficult to feel strong emotions toward my partner. | .283 | .243 | .335 | .716 | .696 | .740 |
| 8. I feel intense emotions toward my partner. * | -.082 | .967 | -.043 | .567 | .877 | .626 |
| 9. When I think about my partner, I feel powerful emotions. * | .039 | .886 | -.068 | .612 | .862 | .634 |
| 10. I have strong feelings toward my partner. * | .017 | .675 | .184 | .637 | .827 | .709 |
| 11. Both the negative and positive feelings that I have toward my partner are rather weak. | .677 | .083 | .002 | .737 | .563 | .593 |
| 12. I do not particularly highly value my partner but I also do not look down on them. | .623 | -.078 | .165 | .697 | .487 | .592 |
| 13. I do not have an extremely positive or an extremely negative opinion of my partner. | .745 | .034 | -.085 | .703 | .495 | .522 |
| 14. I feel neutral toward my partner. | .432 | .146 | .194 | .686 | .598 | .641 |

*Note.* Items with an asterisk (*) are reverse-scored.

**Choice of Final Factor Solution.** Based on the obtained results and theoretical considerations, we decided to retain the one-factor solution. In the multiple-factor solutions, the factors were highly intercorrelated (*r* > .70) and some factors seemed to arise more from methodological characteristics (e.g., item phrasing) than the content of the items. Specifically, in the two-factor solution, reverse-scored items seemed to load onto Factor 2 while the items that combined multiple thoughts (e.g., “neither… nor”) seemed to load onto Factor 1, with the remaining items loading on both factors. In the three-factor solution, once again the reverse-scored items loaded onto Factor 2 and the “double-phrased” items loaded onto Factor 1, with items that used words such as “numb”, “indifferent”, or “a bit” now loading onto Factor 3. As such multiple-factor solutions did not appear theoretically meaningful, we proceeded with the one-factor solution as the most plausible.

***Cronbach’s Alpha Considerations***

The Cronbach’s alpha of all 14 items together was 0.938, and removing any specific item would not have increased it (if-item-deleted Cronbach’s alphas were in the range between 0.931 and 0.936). Removing any specific item from the final 8-item version of the scale would not have increased the Cronbach’s alpha of 0.918 either (if-item-deleted Cronbach’s alphas were in the range between 0.903 and 0.915).

***Convergent and Discriminant Validity***

**Measures.** To test the convergent validity of our new indifference scale, we measured *romantic disengagement* and *disaffection,* each of which should be related to indifference, both conceptually and empirically. We measured romantic disengagement using the 18-item Romantic Disengagement Scale (Barry et al., 2008) that asks participants to rate statements about their feelings and behavior toward their romantic partner on a 7-point scale (1 = Never, 7 = Always). An example item reads: “I don’t feel like dealing with my partner”. Disaffection was measured using the 21-item Marital Disaffection Scale (Kayser, 1996; Kersten, 1990) with items rated on a 4-point scale (1 = Not at all true, 4 = Very true) and an example item reads: “I often feel lonely even though I am with my partner”.

To establish discriminant validity of the new indifference scale, we measured *general partner (d)evaluation*, as described in the main text, to make sure that indifference could be distinguished from general positive or negative evaluations of a romantic partner. We measured *relational boredom* using the full, 15-item version (Harasymchuk & Fehr, 2012) of the scale described in the Pilot Study to show that indifference toward a romantic partner can be distinguished from feelings of boredom in the relationship. Finally, we measured *depression* using the full 7-item version of the subscale used in the Pilot Study to show that our measure captured indifference toward a romantic partner specifically, rather than just general indifference in a person’s life more broadly (with depression as a proxy for this).

**Analytic Strategy.** To test convergent validity, we examined bivariate Pearson correlations between indifference and disengagement, and indifference and disaffection. To test discriminant validity, we examined bivariate Pearson correlations between indifference and relational boredom, indifference and general partner (d)evaluation, and indifference and depression.

**Results.** Correlations among all key variables in Study 1 can be found in Table B3.

***Convergent Validity.*** The bivariate correlations between the scores on the Subjective Interpersonal Indifference Scale and related constructs indicated good convergent validity – as can be seen in Table B3, indifference as measured by the Subjective Interpersonal Indifference Scale had strong positive correlations with romantic disengagement and disaffection.

***Discriminant Validity.*** In terms of discriminant validity, the bivariate correlation between the Subjective Interpersonal Indifference Scale scores and depression was positive but not very strong, as expected, while the bivariate correlations between the Subjective Interpersonal Indifference Scale scores and general partner (d)evaluation, and the Subjective Interpersonal Indifference Scale scores and relational boredom were relatively high (although not higher than .80). To further examine whether the Subjective Interpersonal Indifference Scale and measures of general partner (d)evaluation and relational boredom were capturing separate constructs, we conducted additional factor analyses with the items from these scales (see below for details and results). Importantly, the results of these analyses indicated that items from the Subjective Interpersonal Indifference Scale, from the general partner (d)evaluation measure, and from the relational boredom scale indeed loaded onto separate factors. Furthermore, given the strength of the bivariate correlations between the Subjective Interpersonal Indifference Scale scores and the two scales examined for convergent validity, we also further investigated whether the Subjective Interpersonal Indifference Scale and measures of disengagement and disaffection were tapping into separate constructs. To this end, we first examined the partial correlations between the constructs when general partner (d)evaluation is controlled for, given that overall negativity toward one’s partner could be responsible for these strong associations. Indeed, when general partner (d)evaluation was controlled for, we found that these bivariate correlations became noticeably lower – namely, we found a partial correlation of .35 (*p* < .001) between indifference and disengagement, and .37 (*p* < .001) between indifference and disaffection. To further examine the links between these measures, we conducted additional factor analyses (see below for details and results) which showed that indifference as measured by the Subjective Interpersonal Indifference Scale, romantic disengagement, and disaffection indeed corresponded to separate factors. Thus, the Subjective Interpersonal Indifference Scale showed satisfactory discriminant validity.

**Table B3**

*Means, Standard Deviations, Bivariate Correlations, and Cronbach’s Alpha for Key Variables in Study 1*

| **Variable** | ***M***  **(*SD*)** | **1.** | **2.** | **3.** | **4.** | **5.** | **6.** | **7.** | **8.** | **9.** | **10.** | **11.** | **12.** |
| --- | --- | --- | --- | --- | --- | --- | --- | --- | --- | --- | --- | --- | --- |
| **1. Indifference** | 1.96 (1.11) | .92 |  |  |  |  |  |  |  |  |  |  |  |
| **2. Disengagement** | 2.33 (0.98) | .71*** | .96 |  |  |  |  |  |  |  |  |  |  |
| **3. Disaffection** | 1.56 (0.61) | .80*** | .80*** | .96 |  |  |  |  |  |  |  |  |  |
| **4. General partner (d)evaluation** | 6.20 (1.09) | -.78*** | -.71*** | -.88*** | .96 |  |  |  |  |  |  |  |  |
| **5. Relational boredom** | 2.72 (1.24) | .78*** | .79*** | .89*** | -.84*** | .95 |  |  |  |  |  |  |  |
| **6. Depression** | 0.63 (0.73) | .33*** | .56*** | .42*** | -.39*** | .47*** | .94 |  |  |  |  |  |  |
| **7. Relationship satisfaction** | 5.56 (1.37) | -.75*** | -.75*** | -.87*** | .83*** | -.88*** | -.45*** | .95 |  |  |  |  |  |
| **8. Commitment** | 6.34 (1.07) | -.69*** | -.64*** | -.82*** | .82*** | -.74*** | -.35*** | .74*** | .91 |  |  |  |  |
| **9. Thoughts of breaking up** | 2.15 (1.13) | .55*** | .61*** | .71*** | -.61*** | .70*** | .39*** | -.69*** | -.62*** | .89 |  |  |  |
| **10. Trust** | 5.72 (1.20) | -.66*** | -.69*** | -.84*** | .76*** | -.77*** | -.43*** | .76*** | .69*** | -.71*** | .93 |  |  |
| **11. Life satisfaction** | 4.90 (1.53) | -.44*** | -.53*** | -.53*** | .51*** | -.58*** | -.62*** | .64*** | .47*** | -.49*** | .52*** | .95 |  |
| **12. Stress** | 0.92 (0.77) | .22*** | .49*** | .34*** | -.30*** | .38*** | .71*** | -.35*** | -.26*** | .36*** | -.38*** | -.48*** | .93 |

*Note.* The Cronbach’s alpha for each scale is displayed on the diagonal.

*** *p* < .001

**Additional Factor Analysis: Indifference and Relational Boredom**

We conducted a confirmatory factor analysis to examine whether a one-factor structure (all items loading onto one factor) and a two-factor structure (one factor for indifference, one factor for relational boredom) were a good fit to the data. We conducted this analysis using the package lavaan (v. 0.6-18; Rosseel, 2012) in R (v. 4.4.0; R Core Team, 2024). The R code for these analyses is available in the project OSF folder (<https://osf.io/mfuz2/overview?view_only=f2aa6fef9bf344db803932e6ab33eba7>).

The chi-square test of exact fit for the one-factor model was rejected (*x^2^* (230) = 2449.69; *p* < .001). However, as this test can be sensitive to small misspecifications with large samples, and the model may still be a good/close fit even if not an exact fit to the data, it is important to also consider other indices to form a holistic impression. According to several commonly reported indicators (fit indices, e.g., CFI = 0.80, TLI = 0.78, NNFI = 0.78; SRMR = 0.075; GFI = 0.63) and the root mean square error of approximation (RMSEA = 0.128), the model fit was indeed not satisfactory. For the two-factor model, the chi-square test of exact fit was also rejected (*x^2^* (229) = 1939.06; *p* < .001). According to several commonly reported indicators (fit indices, e.g., CFI = 0.85, TLI = 0.83, NNFI = 0.83; SRMR = 0.066; GFI = 0.68) and the root mean square error of approximation (RMSEA = 0.112), the model fit was still not satisfactory. Therefore, based on the results of an exploratory factor analysis, we ran a model with a three-factor solution: one factor for the indifference items, one item for the reverse-scored boredom items, and one factor for the remaining (non-reversed) boredom items. The chi-square test of exact fit for this model was rejected (*x^2^* (227) = 1126.74; *p* < .001). However, according to several commonly reported indicators (fit indices, e.g., CFI = 0.92, TLI = 0.91, NNFI = 0.91; SRMR = 0.061; GFI = 0.84) and the root mean square error of approximation (RMSEA = 0.082), the model fit was not entirely good, but satisfactory.

Thus, we concluded that the indifference and relational boredom items corresponded to separate factors, with boredom potentially corresponding to two factors that seem to be based on item phrasing.

**Additional Factor Analysis: Indifference and General Partner (D)Evaluation**

We conducted a confirmatory factor analysis to examine whether a one-factor structure (all items loading onto one factor) and a two-factor structure (one factor for indifference, one factor for general partner (d)evaluation) were a good fit to the data. We conducted this analysis using the same procedure as described previously.

The chi-square test of exact fit for the one-factor model was rejected (*x^2^* (65) = 1045.82; *p* < .001). According to several commonly reported indicators (fit indices, e.g., CFI = 0.86, TLI = 0.84, NNFI = 0.84; SRMR = 0.078; GFI = 0.70) and the root mean square error of approximation (RMSEA = 0.160), the model fit was not satisfactory. For the two-factor model, the chi-square test of exact fit was also rejected (*x^2^* (64) = 411.81; *p* < .001). However, according to several commonly reported indicators (fit indices, e.g., CFI = 0.95, TLI = 0.94, NNFI = 0.94; SRMR = 0.040; GFI = 0.89) and the root mean square error of approximation (RMSEA = 0.096), the model fit was not entirely good, but satisfactory.

We therefore concluded that the indifference and overall partner (d)evaluation items corresponded to separate factors.

**Additional Factor Analysis: Indifference, Disengagement, and Disaffection**

We conducted a confirmatory factor analysis to examine whether a one-factor structure (all items loading onto one factor) and a three-factor structure (one factor for indifference, one factor for disengagement, one factor for disaffection) were a good fit to the data. We conducted this analysis using the same procedure as described previously.

The chi-square test of exact fit for the one-factor model was rejected (*x^2^* (1034) = 6345.24; *p* < .001). According to several commonly reported indicators (fit indices, e.g., CFI = 0.77, TLI = 0.76, NNFI = 0.76; SRMR = 0.065; GFI = 0.55) and the root mean square error of approximation (RMSEA = 0.093), the model fit was not satisfactory. For the three-factor model, the chi-square test of exact fit was also rejected (*x^2^* (1031) = 4345.40; *p* < .001). However, according to several commonly reported indicators (fit indices, e.g., CFI = 0.86, TLI = 0.85, NNFI = 0.85; SRMR = 0.055; GFI = 0.71) and the root mean square error of approximation (RMSEA = 0.074), the model fit was not entirely good, but satisfactory.

We therefore concluded that the indifference, disengagement, and disaffection items corresponded to three separate factors.

**Sensitivity Analyses**

Using the same approach as in the Pilot Study, sensitivity analyses revealed that small effect sizes (*f^2^* = 0.013) could be detected in a replication study with 80% power.

**Relationship Security as a Relationship Well-Being Outcome**

As in the Pilot Study, we also tested relationship security as a relationship well-being outcome. We measured relationship security using the same items as in the Pilot Study. Table B4 shows the mean, standard deviation, Cronbach’s alpha, and bivariate correlations of relationship security with other key variables in Study 1.

**Table B4**

*Means, Standard Deviations, Bivariate Correlations, and Cronbach’s Alpha for Key Variables in Study 1*

| **Variable** | ***M***  **(*SD*)** | **1.** | **2.** | **3.** | **4.** | **5.** | **6.** | **7.** | **8.** | **9.** | **10.** | **11.** | **12.** | **13.** |
| --- | --- | --- | --- | --- | --- | --- | --- | --- | --- | --- | --- | --- | --- | --- |
| **1. Indifference** | 1.96 (1.11) | .92 |  |  |  |  |  |  |  |  |  |  |  |  |
| **2. Disengagement** | 2.33 (0.98) | .71*** | .96 |  |  |  |  |  |  |  |  |  |  |  |
| **3. Disaffection** | 1.56 (0.61) | .80*** | .80*** | .96 |  |  |  |  |  |  |  |  |  |  |
| **4. General partner (d)evaluation** | 6.20 (1.09) | -.78*** | -.71*** | -.88*** | .96 |  |  |  |  |  |  |  |  |  |
| **5. Relational boredom** | 2.72 (1.24) | .78*** | .79*** | .89*** | -.84*** | .95 |  |  |  |  |  |  |  |  |
| **6. Depression** | 0.63 (0.73) | .33*** | .56*** | .42*** | -.39*** | .47*** | .94 |  |  |  |  |  |  |  |
| **7. Relationship satisfaction** | 5.56 (1.37) | -.75*** | -.75*** | -.87*** | .83*** | -.88*** | -.45*** | .95 |  |  |  |  |  |  |
| **8. Commitment** | 6.34 (1.07) | -.69*** | -.64*** | -.82*** | .82*** | -.74*** | -.35*** | .74*** | .91 |  |  |  |  |  |
| **9. Thoughts of breaking up** | 2.15 (1.13) | .55*** | .61*** | .71*** | -.61*** | .70*** | .39*** | -.69*** | -.62*** | .89 |  |  |  |  |
| **10. Trust** | 5.72 (1.20) | -.66*** | -.69*** | -.84*** | .76*** | -.77*** | -.43*** | .76*** | .69*** | -.71*** | .93 |  |  |  |
| **11. Relationship security** | 5.96 (1.33) | -.65*** | -.61*** | -.78*** | .77*** | -.74*** | -.36*** | .76*** | .77*** | -.66*** | .79*** | .86 |  |  |
| **12. Life satisfaction** | 4.90 (1.53) | -.44*** | -.53*** | -.53*** | .51*** | -.58*** | -.62*** | .64*** | .47*** | -.49*** | .52*** | .51*** | .95 |  |
| **13. Stress** | 0.92 (0.77) | .22*** | .49*** | .34*** | -.30*** | .38*** | .71*** | -.35*** | -.26*** | .36*** | -.38*** | -.29*** | -.48*** | .93 |

*Note.* The Cronbach’s alpha for each scale is displayed on the diagonal.

*** *p* < .001

When controlling only for overall partner (d)evaluation, indifference as measured by the Subjective Interpersonal Indifference Scale was significantly negatively associated with relationship security (*b* = -0.14; *SE* = 0.05; *t* = -2.77; *p* = .006). When also controlling for relational boredom, indifference as measured by the Subjective Interpersonal Indifference Scale was not significantly associated with relationship security (*b* = -0.01; *SE* = 0.05; *t* = -0.27; *p* = .790).

The results from mediation analyses also showed that feelings of indifference toward one’s partner were associated with lower relationship security via higher relational boredom (indirect effect *ab* = -0.125; *SE* = 0.026; 95% CI [-0.179, -0.076]).

**Criterion Validity Results Controlling for Relational Boredom**

The associations of indifference as measured by the Subjective Interpersonal Indifference Scale and relationship and personal well-being outcomes, above and beyond general partner (d)evaluation and relational boredom, are shown in Table B5.

**Table B5**

*Associations of Subjective Interpersonal Indifference Scale Scores with Relationship and Personal Well-Being, Controlling for General Partner (D)Evaluation and Relational Boredom*

| **Outcome** | ***b*** | ***SE*** | ***t*** | ***p*** |
| --- | --- | --- | --- | --- |
| **Relationship satisfaction** | -0.07 | 0.04 | -1.73 | .085 |
| **Commitment** | -0.09 | 0.04 | -2.21 | .028 |
| **Thoughts of breaking up** | -0.02 | 0.05 | -0.41 | .685 |
| **Trust** | -0.05 | 0.05 | -1.03 | .303 |
| **Life satisfaction** | 0.08 | 0.08 | 0.96 | .336 |
| **Stress** | -0.15 | 0.05 | -3.26 | .001 |

**Supplement C**

**Additional Results Study 2**

This Supplement contains a confirmatory factor analysis of the Subjective Interpersonal Indifference Scale, sensitivity analyses, parallel mediation analyses, and a list of measures included in Study 2 but not used in the present analyses.

**Confirmatory Factor Analysis of the Subjective Interpersonal Indifference Scale**

We conducted a confirmatory factor analysis to examine whether the one-factor structure found for the Subjective Interpersonal Indifference Scale in Study 1 will replicate in Study 2. We conducted this analysis using the package lavaan (v. 0.6-18; Rosseel, 2012) in R (v. 4.4.0; R Core Team, 2024). The R code for these analyses is available in the project OSF folder (<https://osf.io/mfuz2/overview?view_only=f2aa6fef9bf344db803932e6ab33eba7>).

The chi-square test of exact fit for the one-factor model was rejected (*x^2^* (20) = 587.32; *p* < .001). According to several commonly reported indicators (fit indices, e.g., CFI = 0.90, TLI = 0.86, NNFI = 0.86; SRMR = 0.051; GFI = 0.87), the model fit was not particularly good, and not particularly bad. However, the root mean square error of approximation (RMSEA) for the model was 0.170, which was not satisfactory.

To investigate how the model fit might be improved, we examined modification indices. We organized the modification indices in descending order, which allowed us to see, based on the data, which terms could be added to the model to improve the fit the most. It is important to note that these and subsequent analyses must be regarded as exploratory, since we did not make these specific adjustments to the model based on any a-priori determined reasons, but rather we were completely guided by the data themselves. The top suggestion for improving the model according to the modification indices was to add a residual covariance between the two reverse-scored items in the scale. We made this adjustment in a subsequent model.

The chi-square test of exact fit for the one-factor model, with a residual covariance between the reverse-scored items, was rejected (*x^2^* (19) = 241.05; *p* < .001). However, according to several commonly reported indicators (fit indices, e.g., CFI = 0.96, TLI = 0.94, NNFI = 0.94; SRMR = 0.032; GFI = 0.94), this model overall fit the data well. Nevertheless, the RMSEA for the model was still not satisfactory, with a value of 0.109.

We therefore tried to improve model fit once again by looking at modification indices. The top suggestion this time was to add a residual covariance between two of the items (“*I feel a bit numb toward my partner*” and “*I feel a bit indifferent toward my partner*”), so we proceeded by testing a third model with this term added. The chi-square test of exact fit for the one-factor model, with a residual covariance between the reverse-scored items and the above specified items about “feeling a bit numb” and “feeling a bit indifferent”, was rejected (*x^2^* (18) = 139.29; *p* < .001). However, once again, according to several commonly reported indicators (fit indices, e.g., CFI = 0.98, TLI = 0.97, NNFI = 0.97; SRMR = 0.024; GFI = 0.97), this model overall fit the data well. The RMSEA for the model was 0.083, which was not an optimal value but was deemed acceptable.

Based on these results, it seems that a one-factor model overall fits the data relatively well. Namely, based on the modifications suggested by the data, it does not seem that another factor is needed, but rather just residual covariances between specific items that seem to be the result of item wording (i.e., two reverse-worded items and two items that used similar wording with “a bit” and descriptive terms “feeling numb”/”feeling indifferent”).

**Sensitivity Analyses**

Using the same approach as in the Pilot Study and Study 1, sensitivity analyses indicated that a replication study could detect small effect sizes (*f^2^* = 0.008) with 80% power.

**Parallel Mediation Analyses**

We also tested the mediators that were found to be significant mechanisms underlying associations between indifference and relationship outcomes (relational boredom, intimacy, desire for attractive alternatives) and underlying associations between indifference and personal outcomes (relational boredom and intimacy) as parallel mediators, to assess their relative contribution. The results of testing the indirect effects of indifference via the parallel mechanisms on relationship and personal outcomes are shown in Table C1.

Comparing the results to Table 7 in the main text, which shows the results of testing the mechanisms as individual mediators in separate models, the conclusions have largely remained the same. Namely, relational boredom has retained all of its indirect effects, intimacy has retained all but three (on thoughts of breaking up, life satisfaction, and depression), and desire for attractive alternatives has retained all but two (on relationship satisfaction and trust). Overall, despite these discrepancies, it seems that all three mechanisms have a unique role in the examined processes.

**Table C1**

*Results of Testing Indirect Effects of Indifference on Relationship and Personal Outcomes Through Parallel Mechanisms, Controlling for General Partner (D)Evaluation in Study 2*

| **Outcome** | **Indirect effect of indifference via relational boredom** | | | **Indirect effect of indifference via intimacy** | | | **Indirect effect of indifference via desire for attractive alternatives** | | |
| --- | --- | --- | --- | --- | --- | --- | --- | --- | --- |
|  | *ab* | *SE* | 95% CI | *ab* | *SE* | 95% CI | *ab* | *SE* | 95% CI |
| **Relationship satisfaction** | **-0.200** | **0.021** | **[-0.241, -0.161]** | **-0.057** | **0.013** | **[-0.085, -0.033]** | -0.003 | 0.003 | [-0.010, 0.002] |
| **Commitment** | -0.010 | 0.017 | [-0.044, 0.022] | -0.004 | 0.010 | [-0.023, 0.014] | **-0.018** | **0.007** | **[-0.033, -0.005]** |
| **Thoughts of breaking up** | **0.155** | **0.026** | **[0.105, 0.208]** | -0.008 | 0.015 | [-0.038, 0.022] | **0.011** | **0.006** | **[0.002, 0.024]** |
| **Trust** | **-0.095** | **0.022** | **[-0.138, -0.053]** | **-0.033** | **0.014** | **[-0.060, -0.008]** | -0.003 | 0.004 | [-0.012, 0.004] |
| **Life satisfaction** | **-0.204** | **0.034** | **[-0.272, -0.139]** | -0.030 | 0.019 | [-0.068, 0.005] | - | - | - |
| **Stress** | **0.097** | **0.020** | **[0.059, 0.139]** | **0.009** | **0.012** | **[-0.013, 0.032]** | - | - | - |
| **Depression** | **0.101** | **0.019** | **[0.065, 0.138]** | 0.017 | 0.010 | [-0.003, 0.038] | - | - | - |

*Note.* The significant indirect effects are indicated in bold. The dashes indicate that the respective mechanism was not tested as underlying the effects on a given outcome variable.

**Other Measures Included in the Study**

The following measures were included in the study but not used in the present analyses because they will be used to test a different research question: perceived partner indifference, perceived partner boredom, perceived partner responsiveness, support seeking, perceived partner’s general partner (d)evaluation, conflict avoidance, self-esteem, demographic variables.

**Supplement D**

**Additional Results Study 3**

This Supplement contains sensitivity analyses, analyses of additional mechanisms, analyses of relationship security as a relationship well-being outcome, analyses of alternative longitudinal mediation models, parallel mediation analyses, and a list of measures included in Study 3 but not used in the present analyses.

**Sensitivity Analyses**

To adjust for non-independence in our data, we calculated the number of independent observations as *N effective* from the formula: *N*_effective_ = *N * k /* (1 + (*k* - 1) * ICC) (Wiley & Wiley, 2019), where *N* is the number of units that are indeed independent in the data (i.e., couples), *k* is the number of datapoints belonging to each independent unit (i.e., 7 study waves multiplied by two partners in a couple amounts to *k* = 14), and ICC is the intraclass correlation. With the resulting *N effective* as the sample size, we conducted the sensitivity analyses in G*Power v. 3.1.9.7 (Faul et al., 2009) using the same procedure as for the Pilot Study, Study 1, and Study 2. Table D1 shows the results according to the specific outcome variable in the cross-sectional analyses across all study waves.

**Table D1**

*Sensitivity Analyses for Associations of Indifference with Relationship and Personal Well-Being Outcomes in Study 3*

| **Outcome** | **Smallest detectable effect size *f^2^*** | **Power** |
| --- | --- | --- |
| Relationship satisfaction | 0.019 | .80 |
| Commitment | 0.006 | .80 |
| Thoughts of breaking up | 0.014 | .80 |
| Trust | 0.013 | .80 |
| Life satisfaction | 0.007 | .80 |
| Depression | 0.006 | .80 |
| Stress | 0.006 | .80 |

**Analyses of Additional Mechanisms**

We measured several additional mechanisms at different study phases in Study 3. Table D2 shows the descriptive statistics, Cronbach’s alphas, and bivariate correlations between these and other key variables at Study 3 Intake. We conducted the analyses using the analytic approach described in the main text.

***Measures***

**Mechanisms Assessed at Intake.** At Intake, we measured *desire for* *attractive alternatives* using one item about attraction (“How attractive is this person?”) and one item about desire (“How much do you desire this person?”) from Zoppolat and colleagues (2022), responded to on a 7-point scale (1 = Not at all; 7 = Completely). These items were analyzed separately (i.e., one as an indicator of attraction, the other as an indicator of desire) and were only rated by participants who reported that they have an attractive alternative in their life. We also assessed *attention to other domains* at Intake using five one-item measures for each domain respectively (“How much time, energy, dedication you spend on: Your friends/Your family members (other than your partner)/Your work/Your hobbies/Other goals unrelated to the relationship”), responded to on a 7-point scale (1 = No time; 7 = A lot of time). We note that the measures of attention to other domains were developed for this study and despite their face validity, they have not been independently validated as measures of their respective constructs.

**Mechanisms Assessed at Multiple Study Sessions.** *Work engagement* (as another indicator of attention to the work domain) was assessed using 9 items at Intake and 3 items at Follow-ups 3-6 from the Utrecht Work Engagement Scale (Schaufeli et al., 2002; e.g., “I am immersed in my work”), with responses given on a 7-point scale (at Intake: 1 = Not at all, 7 = Completely, with those who do not work or study excluded; at Follow-ups: 0 = Never, 6 = Always).

**Table D2**

*Means, Standard Deviations, Bivariate Correlations, and Cronbach’s Alpha for Key Variables in Study 3 at Intake*

| **Variable** | ***M***  **(*SD*)** | **1.** | **2.** | **3.** | **4.** | **5.** | **6.** | **7.** | **8.** | **9.** | **10.** | **11.** | **12.** | **13.** | **14.** | **15.** | **16.** | **17.** | **18.** |
| --- | --- | --- | --- | --- | --- | --- | --- | --- | --- | --- | --- | --- | --- | --- | --- | --- | --- | --- | --- |
| **1. Indifference** | 1.85  (1.05) | .63 |  |  |  |  |  |  |  |  |  |  |  |  |  |  |  |  |  |
| **2. Relationship satisfaction** | 6.24  (0.82) | -.51*** | .87 |  |  |  |  |  |  |  |  |  |  |  |  |  |  |  |  |
| **3. Commitment** | 6.49  (0.59) | -.36*** | .37*** | .48 |  |  |  |  |  |  |  |  |  |  |  |  |  |  |  |
| **4. Thoughts of breaking up** | 2.25  (1.06) | .40*** | -.49*** | -.31*** | .85 |  |  |  |  |  |  |  |  |  |  |  |  |  |  |
| **5. Trust** | 5.99  (0.77) | -.39*** | .61*** | .31*** | -.52*** | .82 |  |  |  |  |  |  |  |  |  |  |  |  |  |
| **6. Life satisfaction** | 5.61  (1.06) | -.30*** | .46*** | .23*** | -.28*** | .32*** | .86 |  |  |  |  |  |  |  |  |  |  |  |  |
| **7. Stress** | 0.72  (0.58) | .17** | -.16** | -.03 | .19*** | -.12* | -.41*** | .85 |  |  |  |  |  |  |  |  |  |  |  |
| **8. Depression** | 0.36  (0.44) | .21*** | -.11* | -.07 | .11* | -.08 | -.47*** | .63*** | .83 |  |  |  |  |  |  |  |  |  |  |
| **9. Relational boredom** | 2.53  (0.78) | .35*** | -.58*** | -.15** | .30*** | -.33*** | -.33*** | .21*** | .18*** | .82 |  |  |  |  |  |  |  |  |  |
| **10. Attraction to attractive alternative** | 5.51  (1.03) | -.07 | .18** | .05 | -.04 | .18** | .12 | .04 | -.03 | -.03 | - |  |  |  |  |  |  |  |  |
| **11. Desire for attractive alternative** | 2.37  (1.43) | .19** | -.30*** | -.25*** | .23*** | -.17** | -.12 | -.02 | -.04 | .22*** | .16* | - |  |  |  |  |  |  |  |
| **12. Attention to friends** | 4.23  (1.40) | -.02 | .07 | -.01 | .10 | .06 | .13* | .05 | -.02 | -.20*** | .07 | .09 | - |  |  |  |  |  |  |
| **13. Attention to family members** | 4.45  (1.45) | -.01 | .04 | .07 | -.01 | .02 | .04 | -.03 | -.02 | -.09 | .12 | .02 | .26*** | - |  |  |  |  |  |
| **14. Attention to work** | 5.04  (1.53) | -.03 | .01 | .08 | -.02 | -.03 | .10 | .04 | -.05 | -.06 | .01 | -.04 | .11* | -.02 | - |  |  |  |  |
| **15. Attention to hobbies** | 4.49  (1.44) | -.06 | .09 | .01 | -.08 | .05 | .10* | -.15** | -.13* | -.16** | .07 | .00 | .18*** | .04 | -.13* | - |  |  |  |
| **16. Attention to other goals** | 3.84  (1.45) | .01 | -.08 | -.07 | .07 | -.08 | -.06 | .00 | .05 | -.02 | .11 | .13* | .22*** | .21*** | -.07 | .16** | - |  |  |
| **17. Work engagement** | 5.28  (1.20) | -.06 | .06 | .02 | -.08 | .06 | .34*** | -.32*** | -.32*** | -.12* | .00 | .04 | .05 | .07 | .24*** | .08 | .05 | .93 |  |
| **18. General partner (d)evaluation** | 6.76  (0.38) | -.43*** | .68*** | .34*** | -.37*** | .49*** | .35*** | -.13* | -.09 | -.55*** | .05 | -.27*** | .08 | -.05 | .05 | .09 | -.06 | .04 | .83 |

*Note.* For each measure with more than one item, the Cronbach’s alpha is displayed on the diagonal.

* *p* < .05

** *p* < .01

*** *p* < .001

***Results***

The results of testing the indirect effects of indifference via additional mechanisms on relationship outcomes are shown in Table D3. At Intake, the results showed that indifference, controlling for general partner (d)evaluation, was not significantly associated with any of the examined relationship outcomes via attraction to or desire for an attractive alternative, via attention to other domains (friends, family, work, hobbies, or other goals), or via work engagement. Across the available Follow-up waves (waves 3-6), indifference was not significantly associated with the examined relationship outcomes via work engagement, controlling for general partner (d)evaluation.

**Table D3**

*Indirect Effects of Indifference on Relationship Outcomes via Additional Mechanisms, Controlling for General Partner (D)Evaluation in Study 3*

| **Mechanism** | **Relationship satisfaction** | | **Commitment** | | **Thoughts of breaking up** | | **Trust** | |
| --- | --- | --- | --- | --- | --- | --- | --- | --- |
|  | *ab* | 95% CI | *ab* | 95% CI | *ab* | 95% CI | *ab* | 95% CI |
| Attraction to alternative | -0.006 | [-0.024, 0.009] | -0.000 | [-0.007, 0.007] | 0.001 | [-0.011, 0.013] | -0.006 | [-0.025, 0.009] |
| Desire for alternative | -0.008 | [-0.025, 0.004] | -0.007 | [-0.024, 0.004] | 0.010 | [-0.005, 0.034] | -0.001 | [-0.012, 0.008] |
| Dedication to friends | -0.000 | [-0.004, 0.004] | 0.000 | [-0.004, 0.004] | -0.001 | [-0.016, 0.014] | -0.000 | [-0.005, 0.004] |
| Dedication to family | -0.001 | [-0.008, 0.003] | -0.002 | [-0.009, 0.004] | -0.000 | [-0.009, 0.006] | -0.001 | [-0.007, 0.004] |
| Dedication to work | 0.000 | [-0.004, 0.004] | -0.001 | [-0.007, 0.005] | -0.000 | [-0.007, 0.006] | 0.001 | [-0.005, 0.007] |
| Dedication to hobbies | -0.000 | [-0.005, 0.004] | 0.000 | [-0.003, 0.004] | 0.001 | [-0.007, 0.011] | 0.000 | [-0.004, 0.005] |
| Dedication to other goals | 0.001 | [-0.005, 0.008] | 0.001 | [-0.004, 0.007] | -0.001 | [-0.010, 0.006] | 0.001 | [-0.005, 0.008] |
| Work engagement (Intake) | -0.001 | [-0.007, 0.004] | 0.000 | [-0.004, 0.005] | 0.002 | [-0.006, 0.012] | -0.001 | [-0.007, 0.005] |
| Work engagement (Follow-ups 3-6) | -0.001 | [-0.005, 0.001] | -0.002 | [-0.006, 0.001] | -0.000 | [-0.003, 0.002] | -0.001 | [-0.004, 0.001] |

**Relationship Security as a Relationship Well-Being Outcome**

As in the Pilot Study and Study 1, we also tested relationship security as a relationship well-being outcome. We measured relationship security at the six Follow-up sessions using a single face-valid item: “Over the last six months, I felt safe and secure of the bond I have with my partner”, rated on a 7-point scale (1 = Not at all, 7 = Completely). The mean, standard deviation, and bivariate correlations of relationship security with other key variables in Study 3 are shown in Table D4. For the analyses, we applied the same analytic approach as described for the other outcome variables in the main text.

In cross-sectional analyses across the seven study waves, controlling for general partner (d)evaluation, indifference was significantly negatively associated with relationship security (*b* = -0.12; *SE* = 0.02; *t* = -5.22; *p* < .001). Mediation analyses showed that indifference was significantly associated with lower relationship security via higher relational boredom (indirect effect *ab* = -0.031; 95% CI [-0.043, -0.020]), lower intimacy (*ab* = -0.026; 95% CI [-0.048, -0.008]), and higher desire for attractive alternatives (*ab* = -0.010; 95% CI [-0.017, -0.004]).

In longitudinal analyses, controlling for earlier general partner (d)evaluation, earlier indifference was not significantly associated with later relationship security (*b* = -0.02; *SE* = 0.03; *t* = -0.89; *p* = .374). Mediation analyses showed that earlier indifference was significantly associated with lower later relationship security via higher earlier desire for attractive alternatives (*ab* = -0.008; 95% CI [-0.016, -0.002]), but not via earlier relational boredom (*ab* = -0.008; 95% CI [-0.020, 0.004]) or via earlier intimacy (*ab* = -0.016; 95% CI [-0.041, 0.001]). Earlier indifference was also significantly associated with lower later relationship security via higher later relational boredom (*ab* = -0.028; 95% CI [-0.046, -0.011]), but not via later desire for attractive alternatives (*ab* = -0.001; 95% CI [-0.006, 0.003]) or later intimacy (*ab* = -0.025; 95% CI [-0.075, 0.021]).

**Table D4**

*Means, Standard Deviations, Bivariate Correlations, and Cronbach’s Alpha for Key Variables in Study 3 Across Available Timepoints*

| **Variable** | ***M***  **(*SD*)** | **1.** | **2.** | **3.** | **4.** | **5.** | **6.** | **7.** | **8.** | **9.** | **10.** | **11.** | **12.** | **13.** |
| --- | --- | --- | --- | --- | --- | --- | --- | --- | --- | --- | --- | --- | --- | --- |
| **1. Indifference** | 2.04  (1.18) | .73 |  |  |  |  |  |  |  |  |  |  |  |  |
| **2. Relationship satisfaction** | 5.99  (1.03) | -.58*** | .93 |  |  |  |  |  |  |  |  |  |  |  |
| **3. Commitment** | 6.39  (0.72) | -.48*** | .61*** | .68 |  |  |  |  |  |  |  |  |  |  |
| **4. Thoughts of breaking up** | 2.26  (1.06) | .44*** | -.57*** | -.46*** | .85 |  |  |  |  |  |  |  |  |  |
| **5. Trust** | 5.93  (0.84) | -.47*** | .66*** | .48*** | -.59*** | .87 |  |  |  |  |  |  |  |  |
| **6. Life satisfaction** | 5.61  (1.11) | -.36*** | .57*** | .33*** | -.48*** | .47*** | .90 |  |  |  |  |  |  |  |
| **7. Depression** | 0.40  (0.51) | .31*** | -.30*** | -.18*** | .29*** | -.25*** | -.55*** | .88 |  |  |  |  |  |  |
| **8. Stress** | 0.74  (0.65) | .28*** | -.30*** | -.18*** | .33*** | -.27*** | -.48*** | .70*** | .89 |  |  |  |  |  |
| **9. Relational boredom** | 2.75  (0.94) | .51*** | -.73*** | -.43*** | .45*** | -.53*** | -.49*** | .30*** | .32*** | .87 |  |  |  |  |
| **10. Intimacy** | 6.09  (0.98) | -.53*** | .84*** | .61*** | -.53*** | .66*** | .53*** | -.23*** | -.26*** | -.74*** | .85 |  |  |  |
| **11. Desire for attractive alternatives** | 2.64  (1.95) | .26*** | -.31*** | -.32*** | .37*** | -.25*** | -.21*** | .15*** | .18*** | .26*** | -.30*** | - |  |  |
| **12. General partner (d)evaluation** | 6.53  (0.66) | -.52*** | .79*** | .59*** | -.44*** | .61*** | .41*** | -.19*** | -.22*** | -.65*** | .81*** | -.22*** | .92 |  |
| **13. Relationship security** | 6.18  (1.11) | -.43*** | .66*** | .49*** | -.54*** | .59*** | .45*** | -.27*** | -.27*** | -.52*** | .62*** | -.24*** | .59*** | - |

*Note.* For each measure with more than one item, the Cronbach’s alpha is displayed on the diagonal. *** *p* < .001

**Alternative Longitudinal Mechanisms**

As noted in the main text, we also tested an alternative version of the mediation models – namely, we tested whether indifference was associated with *later* mechanisms (controlling for the earlier mechanism and earlier general partner (d)evaluation), and whether these were in turn associated with *later* relationship and personal well-being outcomes (controlling for the earlier mechanism, earlier outcome, earlier general partner (d)evaluation, and earlier indifference).

As a first step, we examined the associations of earlier indifference with later mechanisms. The results showed that earlier indifference was associated with higher later boredom (*b* = 0.06; *SE* = 0.02; *t* = 3.09; *p* = .002) when controlling for earlier levels of boredom and earlier general partner (d)evaluation. The associations of indifference with later desire for attractive alternatives (*b* = 0.02; *SE* = 0.04; *t* = 0.40; *p* = .692) and later intimacy (*b* = -0.04; *SE* = 0.04; *t* = -1.04; *p* = .300) were not significant.

Table D5 shows the results of testing the indirect effects of earlier indifference via later mechanisms with later relationship and personal well-being outcomes. Namely, we found that earlier indifference was associated via higher later relational boredom with later relationship outcomes (lower relationship satisfaction, commitment, and trust, higher thoughts of breaking up) and with later personal outcomes (lower life satisfaction, higher depression and stress).

**Table D5**

*Associations of Earlier Indifference With Later Relationship and Personal Well-Being Outcomes via Later Mechanisms, Controlling for Earlier General Partner (D)Evaluation in Study 3*

| **Outcome** | **Indirect effect of earlier indifference via later relational boredom** | | **Indirect effect of earlier indifference via later intimacy** | | **Indirect effect of earlier indifference via later desire for alternatives** | |
| --- | --- | --- | --- | --- | --- | --- |
|  | *ab* | 95% CI | *ab* | 95% CI | *ab* | 95% CI |
| Relationship satisfaction | **-0.036** | **[-0.059, -0.014]** | -0.028 | [-0.080, 0.024] | -0.001 | [-0.006, 0.004] |
| Commitment | **-0.018** | **[-0.029, -0.007]** | -0.015 | [-0.046, 0.013] | -0.001 | [-0.006, 0.004] |
| Thoughts of breaking up | **0.017** | **[0.006, 0.028]** | 0.010 | [-0.008, 0.033] | 0.001 | [-0.004, 0.006] |
| Trust | **-0.017** | **[-0.029, -0.007]** | -0.014 | [-0.042, 0.012] | -0.001 | [-0.004, 0.003] |
| Life satisfaction | **-0.023** | **[-0.038, -0.009]** | -0.016 | [-0.049, 0.013] | - | - |
| Depression | **0.009** | **[0.004, 0.016]** | 0.008 | [-0.007, 0.027] | - | - |
| Stress | **0.011** | **[0.004, 0.018]** | 0.008 | [-0.008, 0.028] | - | - |

*Note.* The significant indirect effects are indicated in bold. The dashes indicate that the respective mechanism was not tested as underlying the effects on a given outcome variable.

**Parallel Mediation Analyses**

We also tested the mediators that were found to be significant mechanisms underlying associations between indifference and relationship outcomes (relational boredom, intimacy, desire for attractive alternatives) and underlying associations between indifference and personal outcomes (relational boredom and intimacy) as parallel mediators, to assess their relative contribution. We tested the parallel mediation models using data from only those study waves where all mechanisms were measured (i.e., Follow-ups 5 and 6), to allow for a more balanced comparison of their effects. The results of testing the indirect effects of indifference via the parallel mechanisms on relationship and personal outcomes, cross-sectionally and longitudinally (via *earlier* mechanisms), are shown in Table D6.

In terms of cross-sectional results, as compared to the original results in Table 9 in the main text, relational boredom has retained all but two (on commitment and depression) of its indirect effects, intimacy has retained all of its indirect effects, and desire for attractive alternatives has retained only one indirect effect (on commitment).

In terms of longitudinal results, as compared to the original results in Table 9 in the main text, relational boredom has retained one out of its two longitudinal indirect effects (on life satisfaction), intimacy has also retained only one longitudinal indirect effect (on relationship satisfaction), and desire for attractive alternatives has retained three of its longitudinal indirect effects (on relationship satisfaction, commitment, and thoughts of breaking up).

However, it must be noted that the results shown in Table 9 in the main text were obtained using data from more study waves for both relational boredom (measured at all seven study waves) and desire for attractive alternatives (measured at six study waves). Thus, it cannot be ascertained whether the above discrepancies in results were caused by a mechanism not having a unique indirect effect, or simply by lower statistical power in the parallel mediation analyses. We therefore do not draw any substantial conclusions from these specific discrepancies with the original analyses examining each mediator separately, as it seems that, overall, all three mechanisms have unique roles in the examined processes.

**Table D6**

*Results of Testing the Indirect Effects of Indifference Through Parallel Mechanisms on Relationship and Personal Outcomes, Controlling for General Partner (D)Evaluation in Study 3*

| **Outcome** | **Design** | **Indirect effect of indifference via relational boredom** | | **Indirect effect of indifference via intimacy** | | **Indirect effect of indifference via desire for alternatives** | |
| --- | --- | --- | --- | --- | --- | --- | --- |
|  |  | *ab* | 95% CI | *ab* | 95% CI | *ab* | 95% CI |
| Relationship satisfaction | Cross-sectional | **-0.022** | **[-0.038, -0.009]** | **-0.025** | **[-0.044, -0.009]** | -0.007 | [-0.016, 0.000] |
|  | Longitudinal | -0.012 | [-0.029, 0.000] | **-0.015** | **[-0.035, -0.001]** | **-0.023** | **[-0.042, -0.008]** |
| Commitment | Cross-sectional | -0.004 | [-0.012, 0.003] | **-0.008** | **[-0.019, -0.001]** | **-0.011** | **[-0.022, -0.003]** |
|  | Longitudinal | -0.006 | [-0.017, 0.004] | -0.009 | [-0.023, 0.001] | **-0.016** | **[-0.031, -0.004]** |
| Thoughts of breaking up | Cross-sectional | **0.017** | **[0.005, 0.033]** | **0.017** | **[0.005, 0.033]** | 0.006 | [-0.004, 0.018] |
|  | Longitudinal | 0.004 | [-0.007, 0.017] | -0.002 | [-0.016, 0.012] | **0.013** | **[0.000, 0.028]** |
| Trust | Cross-sectional | **-0.010** | **[-0.021, -0.002]** | **-0.012** | **[-0.025, -0.003]** | -0.004 | [-0.014, 0.004] |
|  | Longitudinal | -0.007 | [-0.018, 0.002] | -0.008 | [-0.022, 0.001] | -0.005 | [-0.016, 0.005] |
| Life satisfaction | Cross-sectional | **-0.027** | **[-0.048, -0.010]** | **-0.020** | **[-0.039, -0.006]** | - | - |
|  | Longitudinal | **-0.013** | **[-0.030, -0.000]** | -0.001 | [-0.016, 0.013] | - | - |
| Depression | Cross-sectional | 0.002 | [-0.005, 0.009] | **0.007** | **[0.000, 0.018]** | - | - |
|  | Longitudinal | 0.002 | [-0.007, 0.011] | -0.002 | [-0.013, 0.007] | - | - |
| Stress | Cross-sectional | **0.011** | **[0.002, 0.023]** | 0.002 | [-0.006, 0.012] | - | - |
|  | Longitudinal | 0.003 | [-0.008, 0.015] | 0.000 | [-0.012, 0.013] | - | - |

*Note.* The significant indirect effects are indicated in bold. The dashes indicate that the respective mechanism was not tested as underlying the effects on a given outcome variable.

**Other Measures Included in the Study**

The following measures were included in the study but not used in the present analyses because they will be used to test different research questions: attachment style, emotion regulation strategies, emotion regulation goals, inclusion of other in the self, objective ambivalence, subjective ambivalence, anxiety, need fulfillment, sacrifice-related measures, partner help in regulation of affect, partner help in goal achievement, sexual desire, attitude toward open relationships, open communication, perceived partner ambivalence, self-esteem, self-control, perceived partner responsiveness, ambivalence expectations, personality, conflict, understanding, health, support seeking, attractive alternatives (presence, perception and feelings, cheating with alternative and in general), perceived partner commitment, compersion, new activities, drug use, relationship relevant choice, COVID threat, reasons to distrust partner to take care of needs, guess implicit attitudes toward partner, passionate love, companionate love, work items (connected with colleagues, interfere with work or private life, work fulfilment, work satisfaction), mood, interdependence measures, perceived partner relationship satisfaction, predictability, support provision, relationship behavior (constructive, destructive, approach, avoidance, fluctuations), attempting to change partner, rumination, importance of passionate love, normative expectations about relationships, perceived partner indifference, growth and destiny beliefs, perceived partner positive and negative affect, positive feelings forecast, ambivalence forecast and remembering, perceived partner relational boredom, demographic variables.

In addition to these questionnaires, the larger study contained a computer-based task measuring implicit attitudes toward the partner, a recorded conversation between the partners, and a daily diary phase.

**References**

Barry, R. A., Lawrence, E., & Langer, A. (2008). Conceptualization and assessment of disengagement in romantic relationships. *Personal Relationships*, *15*(3), 297–315. <https://doi.org/10.1111/j.1475-6811.2008.00200.x>

Boateng, G. O., Neilands, T. B., Frongillo, E. A., Melgar-Quiñonez, H. R., & Young, S. L. (2018). Best practices for developing and validating scales for health, social, and behavioral research: A primer. *Frontiers in Public Health*, *6*, 149. <https://doi.org/10.3389/fpubh.2018.00149>

Cattell, R. B. (1966). The scree test for the number of factors. *Multivariate Behavioral Research*, *1*(2), 245–276. <https://doi.org/10.1207/s15327906mbr0102_10>

Đurić, M., Righetti, F., Zoppolat, G., Lohmer, C., & Schneider, I. K. (2025). Mixed signals: Romantic jealousy and ambivalence in relationships. *Emotion, 25*(4), 853–868. [https://doi.org/10.1037/emo0001458](https://psycnet.apa.org/doi/10.1037/emo0001458)

Đurić, M., Righetti, F., Zoppolat, G., Solleiro Saura, C., & Schneider, I. K. (2025). Loves me, loves me not: Perceived romantic partner’s ambivalence is associated with lower personal and relationship well-being. *Emotion*, *25*(5), 1225–1243. [https://doi.org/10.1037/emo0001493](https://psycnet.apa.org/doi/10.1037/emo0001493)

Faul, F., Erdfelder, E., Buchner, A., & Lang, A.-G. (2009). Statistical power analyses using G*Power 3.1: Tests for correlation and regression analyses. *Behavior Research Methods, 41*, 1149–1160. <https://doi.org/10.3758/BRM.41.4.1149>

Harasymchuk, C., & Fehr, B. (2012). Development of a prototype-based measure of relational boredom. *Personal Relationships*, *19*(1), 162–181. <https://doi.org/10.1111/j.1475-6811.2011.01346.x>

Horn, J. L. (1965). A rationale and test for the number of factors in factor analysis. *Psychometrika, 30*(2), 179–185. <https://doi.org/10.1007/BF02289447>

Kayser, K. (1996). The marital disaffection scale: An inventory for assessing emotional estrangement in marriage. *The American Journal of Family Therapy*, *24*(1), 83–88. <https://doi.org/10.1080/01926189508251019>

Kersten, K. K. (1990). The process of marital disaffection: Interventions at various stages. *Family Relations*, *39*(3), 257–265. <https://doi.org/10.2307/584869>

Lovibond, S.H. & Lovibond, P.F. (1995). *Manual for the Depression Anxiety & Stress Scales* (2nd ed.). Psychology Foundation.

O'Connor, B.P. (2024). *EFA.dimensions: Exploratory factor analysis functions for assessing dimensionality*. R package version 0.1.8.4, <https://CRAN.R-project.org/package=EFA.dimensions>.

R Core Team (2024). R: A language and environment for statistical computing. R Foundation for Statistical Computing, Vienna, Austria. <https://www.R-project.org/>

Revelle, W. (2024). *psych: Procedures for psychological, psychometric, and personality research.* Northwestern University, Evanston, Illinois. R package version 2.4.6, <https://CRAN.R-project.org/package=psych>.

Rosseel, Y. (2012). lavaan: An R package for structural equation modeling. *Journal of Statistical Software, 48*(2), 1-36. <https://doi.org/10.18637/jss.v048.i02>

Schaufeli, W. B., Salanova, M., González-Romá, V., & Bakker, A. B. (2002). The measurement of engagement and burnout: A two sample confirmatory factor analytic approach. *Journal of Happiness Studies*, *3*, 71-92. <https://doi.org/10.1023/A:1015630930326>

Velicer, W. F. (1976). Determining the number of components from the matrix of partial correlations. *Psychometrika, 41*(3), 321–327. <https://doi.org/10.1007/BF02293557>

Velicer, W. F., Eaton, C. A., and Fava, J. L. (2000). Construct explication through factor or component analysis: A review and evaluation of alternative procedures for determining the number of factors or components. In R. D. Goffin, & E. Helmes (Eds.), *Problems and solutions in human assessment* (pp. 41-71)*.* Kluwer.

Watkins, M. W. (2018). Exploratory factor analysis: A guide to best practice. *Journal of Black Psychology, 44*(3), 219-246. <https://doi.org/10.1177/0095798418771807>

Wiley, M., & Wiley, J. F. (2019). *Advanced R Statistical Programming and Data Models*. Apress. <https://doi.org/10.1007/978-1-4842-2872-2>

Zoppolat, G., Faure, R., Alonso-Ferres, M., & Righetti, F. (2022). Mixed and conflicted: The role of ambivalence in romantic relationships in light of attractive alternatives. *Emotion*, *22*(1), 81–99. <https://doi.org/10.1037/emo0001055>
